# A stable and replicable neural signature of lifespan adversity in the adult brain

**DOI:** 10.1038/s41593-023-01410-8

**Published:** 2023-08-21

**Authors:** Nathalie E. Holz, Mariam Zabihi, Seyed Mostafa Kia, Maximillian Monninger, Pascal-M. Aggensteiner, Sebastian Siehl, Dorothea L. Floris, Arun L. W. Bokde, Sylvane Desrivières, Herta Flor, Antoine Grigis, Hugh Garavan, Penny Gowland, Andreas Heinz, Rüdiger Brühl, Jean-Luc Martinot, Marie-Laure Paillère Martinot, Dimitri Papadopoulos Orfanos, Tomáš Paus, Luise Poustka, Juliane H. Fröhner, Michael N. Smolka, Nilakshi Vaidya, Henrik Walter, Robert Whelan, Gunter Schumann, Andreas Meyer-Lindenberg, Daniel Brandeis, Jan K. Buitelaar, Frauke Nees, Christian Beckmann, IMAGEN Consortium, Jean-Luc Martinot, Marie-Laure Paillère Martinot, Juliane H. Fröhner, Michael N. Smolka, Henrik Walter, Tobias Banaschewski, Andre F. Marquand

**Affiliations:** 1 Donders Institute for Brain, Cognition and Behavior, Radboud University Nijmegen, Nijmegen, the Netherlands; 2 Department for Cognitive Neuroscience, Radboud University Medical Center Nijmegen, Nijmegen, the Netherlands; 3 Institute of Medical Psychology and Medical Sociology, University Medical Center Schleswig Holstein, Kiel University, Kiel, Germany; 4 Department of Child and Adolescent Psychiatry and Psychotherapy, Central Institute of Mental Health, Medical Faculty Mannheim, Heidelberg University, Mannheim, Germany; 5 MRC Unit for Lifelong Health & Ageing, University College London (UCL), London, UK; 6 Department of Psychiatry, University Medical Center Utrecht, Utrecht, the Netherlands; 7 Methods of Plasticity Research, Department of Psychology, University of Zurich, Zurich, Switzerland; 8 Discipline of Psychiatry, School of Medicine and Trinity College Institute of Neuroscience, Trinity College Dublin, Dublin, Ireland; 9 Social, Genetic and Developmental Psychiatry Centre, Institute of Psychiatry, Psychology & Neuroscience, King’s College London, London, UK; 10 Institute of Cognitive and Clinical Neuroscience, Central Institute of Mental Health, Medical Faculty Mannheim, Heidelberg University, Mannheim, Germany; 11 Department of Psychology, School of Social Sciences, University of Mannheim, Mannheim, Germany; 12 NeuroSpin, CEA, Université Paris-Saclay, Gif-sur-Yvette, France; 13 Departments of Psychiatry and Psychology, University of Vermont, Burlington, VT USA; 14 Sir Peter Mansfield Imaging Centre School of Physics and Astronomy, University of Nottingham, University Park, Nottingham, UK; 15 Department of Psychiatry and Psychotherapy CCM, Charité—Universitätsmedizin Berlin, Corporate Member of Freie Universität Berlin, Humboldt-Universität zu Berlin, and Berlin Institute of Health, Berlin, Germany; 16 Physikalisch-Technische Bundesanstalt (PTB), Braunschweig and Berlin, Berlin, Germany; 17 Institut National de la Santé et de la Recherche Médicale, INSERM U1299 ‘Developmental Trajectories & Psychiatry’; Université Paris-Saclay, Ecole Normale supérieure Paris-Saclay, CNRS, Centre Borelli, Gif-sur-Yvette, France; 18 Institut National de la Santé et de la Recherche Médicale, INSERM U1299 ‘Developmental Trajectories & Psychiatry’; Université Paris-Saclay, Ecole Normale supérieure Paris-Saclay, CNRS, Centre Borelli, Gif-sur-Yvette; and AP-HP.Sorbonne Université, Department of Child and Adolescent Psychiatry, Pitié-Salpêtrière Hospital, Paris, France; 19 Departments of Psychiatry and Neuroscience and Centre Hospitalier Universitaire Sainte-Justine, University of Montreal, Montreal, Quebec Canada; 20 Departments of Psychiatry and Psychology, University of Toronto, Toronto, Ontario Canada; 21 Department of Child and Adolescent Psychiatry, Centre for Psychosocial Medicine, Heidelberg University, Heidelberg, Germany; 22 Department of Child and Adolescent Psychiatry and Psychotherapy, University Medical Centre Göttingen, Göttingen, Germany; 23 Department of Psychiatry and Psychotherapy, Technische Universität Dresden, Dresden, Germany; 24 PONS-Centre, Department of Psychiatry and Clinical Neuroscience, CCM, Charite University Medicine, Berlin, Germany; 25 School of Psychology and Global Brain Health Institute, Trinity College Dublin, Dublin, Ireland; 26 Centre for Population Neuroscience and Precision Medicine (PONS), Institute for Science and Technology of Brain-inspired Intelligence (ISTBI), Fudan University, Shanghai, China; 27 Department of Psychiatry and Psychotherapy, Central Institute of Mental Health, Medical Faculty Mannheim, Heidelberg University, Mannheim, Germany; 28 Department of Child and Adolescent Psychiatry and Psychotherapy, University Hospital of Psychiatry Zurich, University of Zurich, Zurich, Switzerland; 29 Neuroscience Center Zurich, University of Zurich and ETH Zurich, Zurich, Switzerland; 30 Karakter Child and Adolescent Psychiatry University Center, Nijmegen, The Netherlands; 31 Centre for Functional MRI of the Brain, University of Oxford, Oxford, UK; 32 Department of Neuroimaging, Institute of Psychiatry, Psychology & Neuroscience, King’s College London, London, UK; 33 Institut National de la Santé et de la Recherche Médicale, INSERM U A10 ‘Trajectoires développementales en psychiatrie’, Université Paris-Saclay, Ecole Normale Supérieure Paris-Saclay, CNRS, Centre Borelli, Gif-sur-Yvette, France

**Keywords:** Stress and resilience, Predictive markers

## Abstract

Environmental adversities constitute potent risk factors for psychiatric disorders. Evidence suggests the brain adapts to adversity, possibly in an adversity-type and region-specific manner. However, the long-term effects of adversity on brain structure and the association of individual neurobiological heterogeneity with behavior have yet to be elucidated. Here we estimated normative models of structural brain development based on a lifespan adversity profile in a longitudinal at-risk cohort aged 25 years (*n* = 169). This revealed widespread morphometric changes in the brain, with partially adversity-specific features. This pattern was replicated at the age of 33 years (*n* = 114) and in an independent sample at 22 years (*n* = 115). At the individual level, greater volume contractions relative to the model were predictive of future anxiety. We show a stable neurobiological signature of adversity that persists into adulthood and emphasize the importance of considering individual-level rather than group-level predictions to explain emerging psychopathology.

## Abstract (web summary)

In a birth cohort, Holz et al. found widespread structural brain changes at the age of 25 years as a function of adversity. This pattern was replicated at the age of 33 years and in another cohort. Individual-level volume reductions on top of this pattern predicted anxiety.

## Main

Encountering environmental adversities may increase the risk of developing psychiatric disorders in adulthood^1,2^. Through adaptations in the regulation of emotional, cognitive and behavioral processes, individuals strive to cope with challenging environmental conditions. However, this can be maladaptive and thereby increase the risk for psychopathology (for example, ref. ^3^). Despite the clear adversity-induced vulnerability to developing psychiatric disorders, the neurobiological mechanisms underlying this association have remained elusive for several reasons. First, the focus on regions of interest to replicate previous findings of neurobiological correlates of this association and gain a better understanding of them has led to increased attention on the limbic system and its regulatory control regions (for example, refs. ^4–6^). However, concentrating only on localized effects may neglect interindividual differences in structural or functional organization (for example, individual variation in the spatial distribution of different regions), and may overlook substantial whole-brain changes due to widespread restructuring during development^7–10^. Indeed, meta-analyses have synthesized evidence on a whole-brain level and confirmed a convergence of developmental risk factors in key regions of affective and cognitive regulatory processing, both within and beyond the limbic system^11–15^. Although these results shed light on the interplay between adversities and neural plasticity, they may, however, be influenced by between-study heterogeneity including differences in assessments, participants and statistical analyses.

Second, the abundance of inconsistent findings, even when testing associations between the same adversity and the same brain outcome^4^, has impeded the discovery of the exact neurobiological mechanisms. One reason for such inconsistent findings pertains to the dominance of studies designed to test differences in terms of group means (that is, averages). In such studies, individual-level variability is obscured by averaging across groups. As such, high subject variability can result in null findings because opposing effects observed at the individual subject level may cancel each other out. Likewise, contradictory findings can arise across studies, for instance, an increase or a decrease in brain volume associated with adversities (as reviewed by, for example, refs. ^4,16^).

Third, the impact of adversity during development on brain structure and function has predominantly been investigated without taking into account the typical patterns of brain growth and development. This poses a challenge in identifying neurobiological alterations amidst individual age and sex-specific trajectories and hinders the discovery of the precise mechanisms underlying adaptation. For instance, if the volume of a region follows an inverted u-shaped trajectory that reaches its peak in youth, then a larger volume in childhood associated with adversity would suggest accelerated maturation. In contrast, the same pattern observed after adolescence would suggest delayed maturation (for example, ref. ^4^). This issue could be addressed by referencing adversity-related effects to normative brain growth charts^17–20^, which, akin to pediatric growth charts, enable the quantification of individual variation with respect to population percentiles.

Fourth, adversities are by nature correlated; an individual growing up in a poor environment is more likely to encounter family adversity and stressful life events over their lifetime^21^. Therefore, particularly in adulthood, studies assessing the effect of single adversity are difficult to interpret, as any brain differences may reflect multiple stressors with potentially distinct neural effects^4^.

Fifth, longitudinal studies investigating the enduring effects of adversity on brain structure are limited, with a few exceptions^22,23^. Furthermore, the scarcity of studies examining the effect of adversity on brain development has impeded the possibility to probe whether neurobiological correlates of environmental adversity are stable. Although initial studies in children and adolescents indicate that these correlates may be stable over development^24–26^, it is premature to draw strong conclusions, considering the different methodologies applied in these and the lack of replication in independent cohorts.

Thus, there is a need for predictive mechanistic models that can account for the long-lasting effects of lifespan adversity on a whole-brain level while simultaneously accommodating the interindividual neurobiological heterogeneity.

**Fig. 1:**
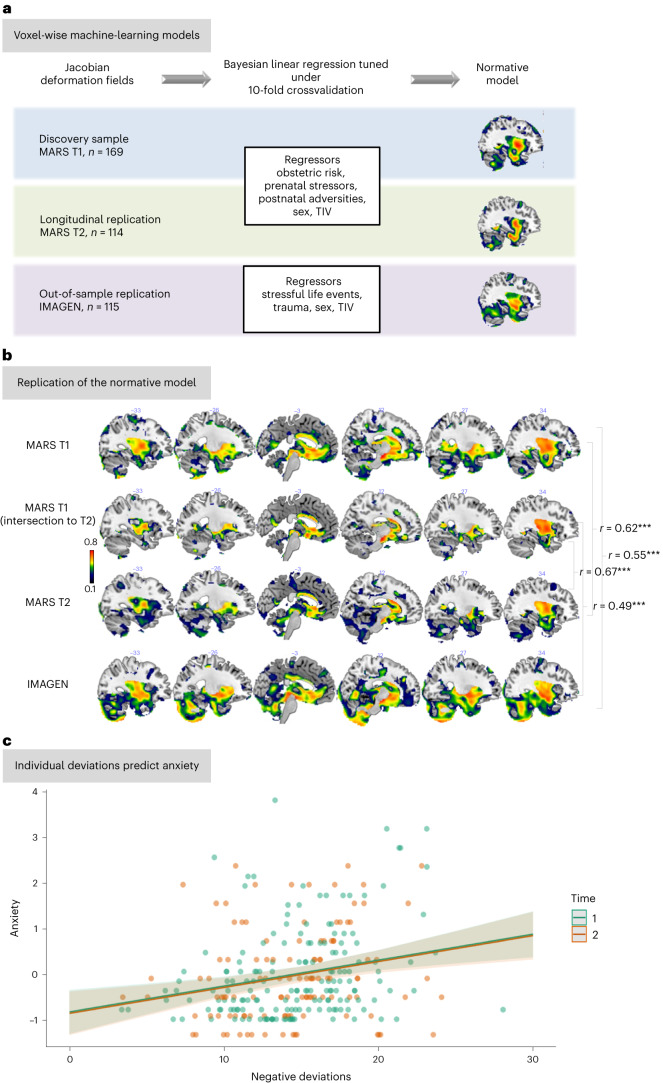
Normative models based on adversity. **a**, Methodological approach—we estimated a voxel-wise normative model of the development of JDs of the deformation fields, which quantifies the degree of volumetric expansion or contraction required to match each sample to the template used in registration (outcome), based on lifetime adversities, TIV and sex as predictors in the MARS sample when participants were 25 years old. Therefore, we performed a Bayesian linear regression under tenfold cross-validation. We replicated this normative model in the MARS sample at the age of 33–34 years and using the sociodemographically similar IMAGEN subsample aged 22 years with comparable adversity measures. **b**, Spatial representation of the voxel-wise Pearson correlations (two-sided) between the true morphometric changes of the JDs and the predicted values in the normative models built on adversities, sex and TIV. First panel: normative model of MARS participants (*n* = 169) at the age of 25 years (T1; brain regions listed in Supplementary Table 1); second panel: normative model of MARS participants (*n* = 114) at the age of 25 years (intersection of participants from the 25-year and 33-year assessments); third panel: replication of the normative model of MARS individuals (*n* = 114) scanned again at the age of 33–34 years (T2, brain regions listed in Supplementary Table 3); fourth panel: replication of this model in a subsample (*n* = 115) of the IMAGEN cohort (22 years, brain regions listed in Supplementary Table 5). **c**, Negative deviations per subject, that is, more volume contractions than expected from the normative model, predicted anxiety at 25 years (T1, *β* coefficient = 0.07, standard error (s.e.) = 0.02, *P* = 0.00006 (two-sided), *η*^2^ = 0.10) and at 33 years (T1 and T2, *β* coefficient = 0.06, s.e. = 0.02, *P* = 0.0005 (two-sided), *η*^2^ = 0.06). Triple asterisks indicate that the Pearson correlation was significant at *P* < 0.001, two-sided. The shaded area represents the 95% confidence interval of the predicted values. Source data

**Extended Data Fig. 1:**
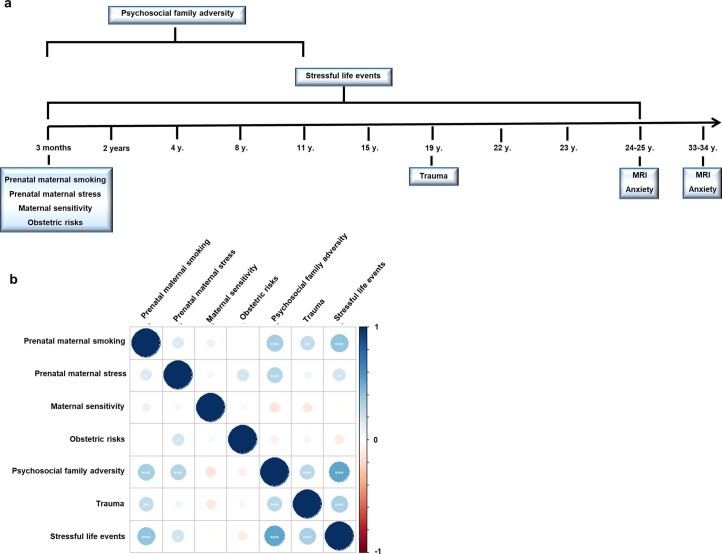
Study design. **a**, MARS assessments, **b**, Spearman correlation plot (* p < 0.05, ** p < 0.01, *** p < 0.001, two-sided, not adjusted for multiple comparisons).

To this aim, we applied a voxel-wise normative modeling approach, which allows us to quantify centiles of variation of adversity effects across the population in a normative model and draw inferences regarding the association of behavior with neurobiological heterogeneity at the individual level, that is, beyond group-average effects^27^. We capitalized on a well-phenotyped adult at-risk cohort followed since birth ("Mannheim Study of Children at Risk" (MARS)). The MARS cohort underwent 11 assessment waves, in which developmental risk factors—including prenatal, perinatal and psychosocial adversities (depicted in Extended Data Fig. 1a)—were prospectively acquired up to adulthood. Importantly, previous research has already established a link between these risks and neurobiological alterations (for example, refs. ^5,28–30^). Using this dataset, we built a spatially precise normative model capturing the long-term neural adaption to these adversities. Furthermore, we tested the stability of this normative model over time by leveraging neuroimaging data at two time points in adulthood in the MARS cohort. We also tested the model in an independent cohort, namely in a population-based subsample from the IMAGEN ("Reinforcement-related behaviour in normal brain function and psychopathology") cohort that has a similar sociodemographic background and assessment of adversities as the MARS cohort (Fig. 1a). To evaluate whether adversity-specific alterations may indicate a delay or an acceleration of development, we estimated a normative model of age-related volumetric changes across development in a very large compilation of data, comprising publicly available datasets in addition to the MARS and IMAGEN cohorts, to serve as a reference. Finally, given that individual variability on top of normative models has shown superior predictive power regarding psychopathology when compared to unmodeled data^31,32^, we investigated how individual deviations from normative brain trajectories are associated with psychopathology. Here we did not formulate any directional hypotheses, given that previous research has not provided consistent evidence for brain-behavior relationships, even for well-investigated limbic brain regions^16,33^ and the lack of models of normative brain development that would allow developmentally-specific predictions.

## Results

### A developmentally stable signature of adversity

First, we mapped the associations between exposure to lifetime adversity and changes in brain structure at the age of 25 years. We quantified brain structure in terms of regional volumetric expansion or contraction using the Jacobian determinants (JDs) of the deformation fields derived from a nonlinear registration (Methods). We then fit voxel-wise normative models to predict these measures based on a broad panel of adversities (Table 1). These seven adversities were mainly prospectively assessed and covered the prenatal period (prenatal maternal smoking and stress), the perinatal period (obstetric risks at birth) and the postnatal period (psychosocial adversities, including lower early maternal care, an adverse family environment over a period of 11 years, childhood traumatic events and stressful life events from birth to adulthood). Details on these measures are described in Methods. To balance the differing severity across adversities, we binned each adversity and the number of subjects in each adversity category, as depicted in Supplementary Fig. 1. However, our results were not sensitive to this choice (Sensitivity analyses).

**Table 1:** Sample characteristics

Characteristics	Age(s) of assessment	Range	Mean (s.d.)
Maternal smoking during pregnancy	3 months	0–2	0.36 (0.71)
Prenatal maternal stress	3 months	0–8	2.74 (1.81)
Maternal sensitivity^a^	3 months	0–1015	503.43 (176.56)
Obstetric adversity	3 months	0–4	0.95 (0.98)
Psychosocial family adversity	3 months, 2 years, 4–5 years, 8 years, 11 years	0–10	3.5 (2.41)
Life events	3 months, 2 years, 4–5 years, 8 years, 11 years, 15 years, 19 years, 22 years, 23 years, 25 years	24–116	53.04 (16.59)
Childhood trauma	19 years	25–63	29.60 (5.92)
Anxiety problems, T1	25 years	0–14	2.82 (2.73)
Anxiety problems, T2	33–34 years	0–10	3.24 (2.44)

^a^Raw values, *z*-transformed variable used for the analyses.

The normative model revealed a widespread morphometric signature of adversity at the age of 25 years, that is, regions where morphometric changes could be accurately predicted by the seven adversity scores as listed above. This distributed pattern extended beyond previously identified regions of interest—such as the hippocampus, amygdala, basal ganglia and ventromedial prefrontal (vmPFC) and anterior cingulate cortex (ACC)—and additionally included the thalamus, middle and superior frontal gyri, occipital gyrus and precentral gyrus (Fig. 1b, first and second panels), among other regions (Supplementary Tables 1 and 2).

Notably, this signature was stable over time, as it was evident in the same participants at 33 years (Fig. 1b, third panel, and Supplementary Tables 3 and 4) and was replicated in an independent sample (Fig. 1b, fourth panel, and Supplementary Tables 5 and 6).

#### Specific effects

**Fig. 2:**
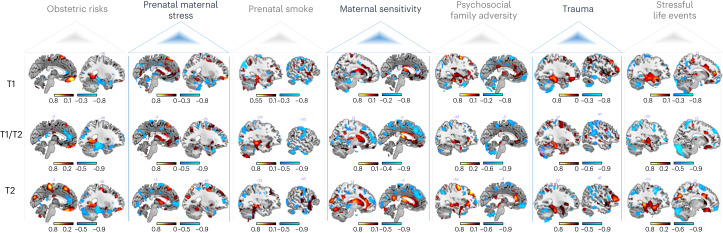
Spatial representation of the structure coefficients. These indicate the correlation between each adversity and the predicted morphometric changes of the JDs of deformation fields. Shown is one sample slice of the top 2% of the voxel-wise contribution for the positive (hot colors) and the negative associations (cold colors). Top, 169 MARS participants at the age of 25 years (T1); middle, 114 MARS participants at the age of 25 (intersection of participants from the 25-year (T1) and 33-year assessments (T2)); and bottom, 114 MARS individuals scanned again at the age of 33 years (T2). More slices of these structure coefficients and the structure coefficients of the IMAGEN sample are shown in Extended Data Fig. 2 and Supplementary Fig. 4, respectively, and all brain regions are listed in Supplementary Tables 7–20. Source data

**Extended Data Fig. 2:**
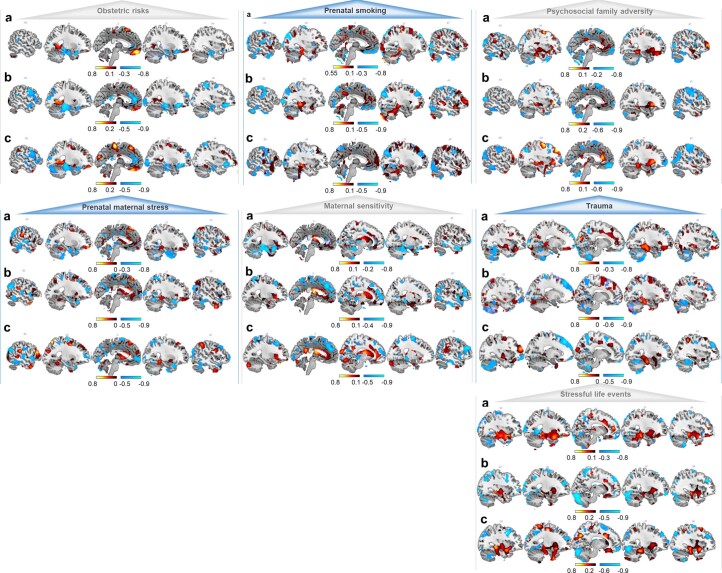
MARS structure coefficients. Spatial representations of the top 2% of the voxel-wise contribution of each adversity on predicted morphometric changes identified based on structure coefficients for **a**, 169 MARS participants at the age of 25, **b**, 114 MARS participants at the age of 25 (intersection of participants from the 25 year and 33 year- assessment), **c**, 114 MARS individuals scanned again at the age of 33 years.

To better understand the contribution of each adversity to the overall adversity pattern, we used structure coefficients. These capture the unique impact of each adversity on the morphometric changes predicted by the normative model, independent of the other predictors, and are particularly useful in situations of collinearity compared to regression coefficients^34^. These indicated that, for instance, in limbic areas specific adversities elicited distinct effects (Fig. 2, more slices depicted in Extended Data Fig. 2 and Supplementary Tables 7–20), as described below.

We also quantified the overlap between the effects of different adversities directly. The highest whole-brain overlap was observed for the structure coefficient maps of the psychosocial risks, that is, family adversity, trauma and stressful life events, with dice coefficients up to 0.54 (Supplementary Table 21). In general, the dice coefficients indicated a low to moderate overlap between the brain patterns associated with different adversities. These findings suggest the emergence of adversity- and region-specific volumetric expansions or contractions with increasing adversity. The average dice coefficients of 0.23 for prenatal smoke exposure and 0.24 for obstetric adversity showed that these had the least overlap with the structure coefficient maps of other adversities. For obstetric adversity, we observed volume expansions in the ventromedial orbitofrontal cortex (vmOFC) and volume contractions in the ACC. For prenatal smoke exposure, a different pattern emerged, with expansions in the hippocampus and contractions in the postcentral and occipital gyrus. By contrast, the highest mean dice coefficient was found for psychosocial family adversity (0.36), with expansions in subcortical limbic areas and contractions in the vmOFC.

#### Multivariate effects

**Fig. 3:**
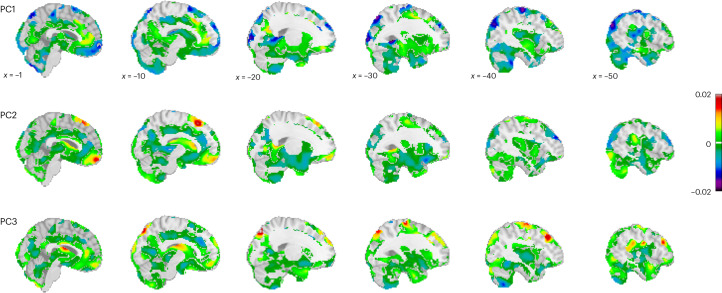
Predictions of how brain morphometry (Jacobian determinants of deformation fields) changes as a function of adversity. Spatial representation of the voxel-wise normative models for each of the PCs based on four sampling points spanning the range of the PC loadings. The panels show the *β* values (slopes) depicting the change, with warm colors indicating a volume expansion and cold colors indicating a volume contraction with increasing adversity. The adversity maps are shown relative to the baseline model. Source data

**Extended Data Fig. 3:**
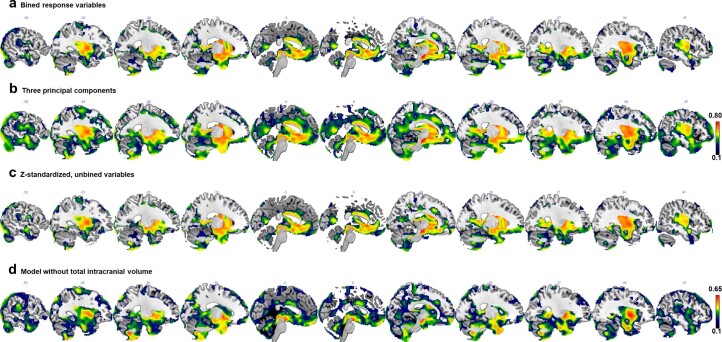
Sensitivity analyses. Spatial representations of the voxel-wise reference models built on **a**, adversity, total intracranial volume (TIV), and sex; **b**, on three principal components, TIV, and sex, and **c**, on z-standardized unbinned adversity scores, TIV, sex; **d**, on binned adversity scores and sex (without total intracranial volume), via Bayesian linear regression under 10-fold crossvalidation in n = 169 MARS participants at the age of 25.

**Extended Data Fig. 4:**
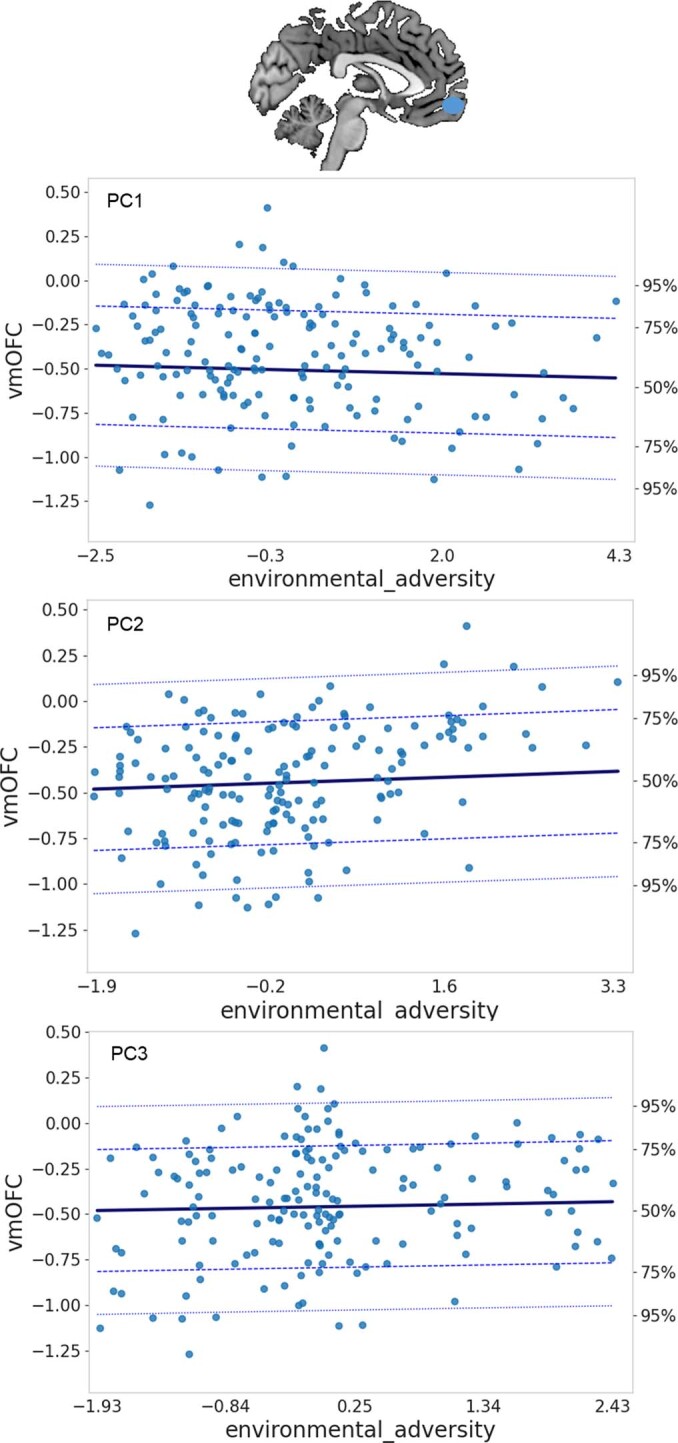
Trajectory of the vmOFC. Structure alterations as a function of the three principal components.

To better understand the effects of multiple adversities in combination, we conducted a principal component (PC) analysis to summarize the primary sources of variance within the set of adversities. This is important because adversities are known to be correlated, as was also the case in our sample (Extended Data Fig. 1b). We, therefore, re-estimated the model using three components that account for two-thirds of the cumulative variance in the adversity scores (Extended Data Fig. 3a,b depict the strong correspondence to the original normative model). Next, we mapped morphological patterns associated with variation across these adversity factors (that is, PCs) by plotting the predictions from these models across the range of adversities spanned by these components. The PC loadings are shown in Supplementary Table 22, and their associated morphometric patterns are shown in Fig. 3. The first PC (PC1) reflected lifespan psychosocial family adversities and prenatal smoking and was mostly related to cortical and subcortical volume contractions, particularly in the vmOFC and the medial frontal gyrus, precentral and postcentral gyrus, frontal pole/middle frontal gyrus, superior frontal gyrus, the inferior temporal gyrus, the caudate, as well as in the occipital lobe. Slight volume expansions were only observed in small clusters in the vmPFC, paracingulate gyrus, superior frontal gyrus and the lateral frontal pole. By contrast, a more balanced picture of region-specific expansions and contractions emerged with regard to PC2, representing early exposures to obstetric risk and prenatal maternal stress. Contractions were most pronounced in the lateral frontal pole, frontal orbital cortex, inferior and superior frontal gyrus, middle temporal gyrus, fusiform gyrus, hippocampus, supramarginal gyrus, parietal and occipital gyrus, and expansions were most pronounced in the medial frontal pole, including the vmOFC, the caudate and the superior frontal gyrus. Similarly for PC3, representing early maternal sensitivity, contractions, particularly in the paracingulate gyrus, supramarginal gyrus, insula, precuneus, lateral occipital gyrus, precentral gyrus, supplementary motor cortex, and expansions in the middle/superior frontal gyrus, the vmPFC/perigenual anterior cingulate, vmOFC, precentral gyrus, the angular gyrus and the thalamus, are revealed. Interestingly, we observed opposing trajectories in, for example, the vmOFC, with volume contractions as a function of higher scores on PC1 and volume expansions as a function of higher scores on PC2 and PC3 (Extended Data Fig. 4).

#### Sensitivity analyses

We performed multiple sensitivity analyses to confirm the robustness of our results. These primarily entailed changes to the specific predictors to which we fit normative models. For instance, we predicted JD development based on adversity factors, which did not change the model (as mentioned above; Extended Data Fig. 3b).

The same pattern of neural alterations emerged when feeding unbinned (raw scores; Methods) adversities (*r* = 0.97, *P* < 0.001; Extended Data Fig. 3c). Likewise, we repeated the whole analysis pipeline excluding total intracranial volume (TIV), which did not change any of the results (see Extended Data Fig. 3d for the pattern of the normative model).

**Extended Data Fig. 5:**
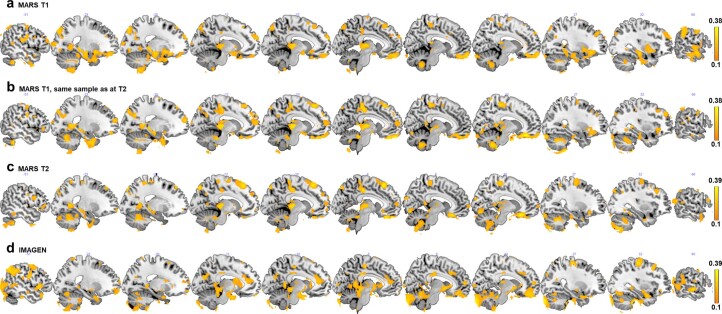
Normative models based on adversity. Spatial representation of the voxel-wise reference models built on adversity only via Bayesian linear regression under 10-fold crossvalidation. **a**, 169 MARS participants at the age of 25; **b**, 114 MARS participants at the age of 25 (intersection of participants from the 25 year and 33 year- assessments); **c**, 114 MARS individuals scanned again at the age of 33 years; **d**, replication of this model in a subsample (n = 115) with similar sociodemographics of the IMAGEN cohort.

Furthermore, models based on only ‘lifetime adversity’ confirmed the stable and replicable pattern particularly in the ventromedial prefrontal cortex, and precuneus, posterior and anterior cingulate, superior frontal and limbic regions such as the hippocampus (Extended Data Fig. 5), although the accuracy of the normative models for predicting brain structure was somewhat lower when sex was excluded from the model; this is expected given the strong influence of sex on brain volume^35^.

In addition, we excluded obstetric adversities given these are of a qualitatively different nature compared to the other risk factors. The normative model remained unchanged and correlated highly with the original normative model (*r* = 0.95, *P* > 0.001). Moreover, we replaced JDs with modulated gray matter volume as an outcome (Supplementary Information and Supplementary Tables 23 and 24); this yielded similar results, and the normative models correlated significantly with each other (*r* = 0.25, *P* < 0.001).

Given the recent discussion on differences emerging from prospective and retrospective self-report measures, we set up separate models that included retrospectively reported trauma, until the age of 18 years and a prospectively assessed life events score until the age of 19 years. Interestingly, the effects of both adversities showed commonalities in the (anterior) cingulate, insula, thalamus, frontal pole, precuneus, lateral orbitofrontal cortex and hippocampus, whereas retrospectively assessed trauma was related to the posterior cingulate (self-reflection). The effects of life events were more widespread and showed stronger effects in a cluster comprising the amygdala, the temporal pole as well as the orbital frontal cortex and the basal ganglia.

#### Normative model of age-related development

**Fig. 4:**
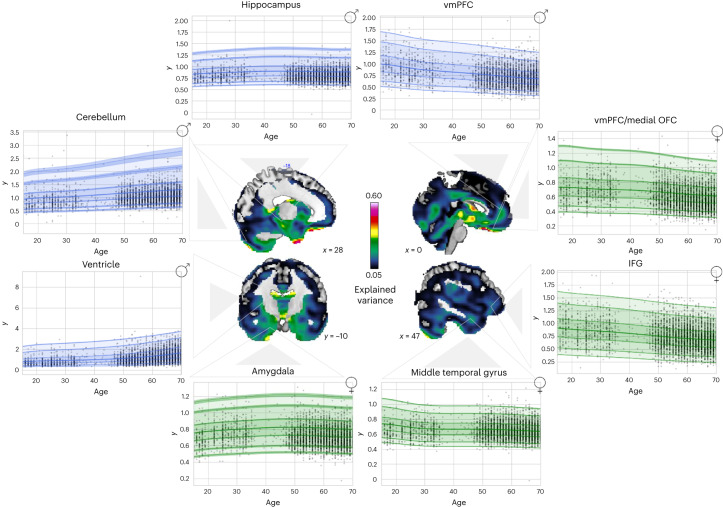
Age-related JD development. The data were split into training and test sets, and normative models were fit to predict JD development based on age, sex and site by using a warped Bayesian linear regression model. Explained variance in the full test set and visualizations for all regional age-related trajectories (green = females and blue = males; centiles of variation correspond to 1%, 5%, 25%, 50%, 75%, 95% and 99%) are depicted. For each region, the trajectory for only one sex is shown, but trajectories were similar for both sexes. Source data

**Extended Data Fig. 6:**
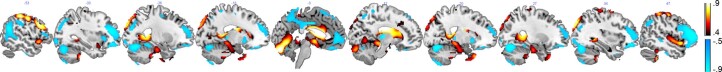
Structure coefficient of age-related development. Spatial representation of the top 2% of the voxel-wise contribution of the correlation of age with predicted morphometric changes.

To interpret adversity-specific effects as indicating acceleration or delay of maturation, we assembled a large dataset from publicly available data repositories and merged it with the MARS and IMAGEN cohorts, containing 19,759 individuals in total across nine scan sites (see Supplementary Tables 25 and 26 for demographics). Based on this, we estimated the developmental trajectory of age-related volume contractions and expansions from 8 to 97 years of age. To this end, we fit normative models to predict JDs development based on age, sex and scanning site, as done previously^18^. The resulting model explained up to 60% of the variance in morphometric development (Fig. 4 and Supplementary Tables 27 and 28). Age-related expansions were observed in limbic subcortical areas, the medial part of the cerebellum and the ventricles, among other regions; age-related contractions were observed in, for example, frontal regions including the vmPFC/vmOFC, the ACC and the inferior frontal gyrus (Extended Data Fig. 6 and Supplementary Tables 29 and 30).

### Individual deviations from the normative model

**Extended Data Fig. 7:**
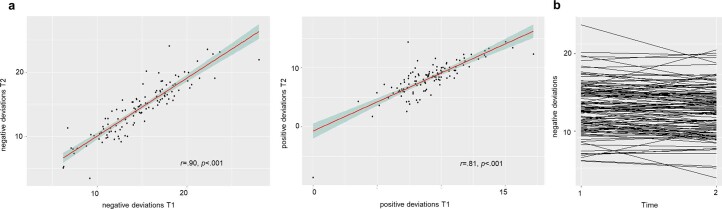
Individual deviations. **a**, Pearson correlations (two-sided) between time points for negative (volume contractions, left, exact p = 2.372827e-41) and for positive deviations (volume expansions, right, exact p = 4.159398e-27) at the 25-year assessment (T1) and 33-year assessment (T2) with 95% confidence intervals, **b**, spaghetti plot showing the change of the negative deviations between both time points.

**Extended Data Fig. 8:**
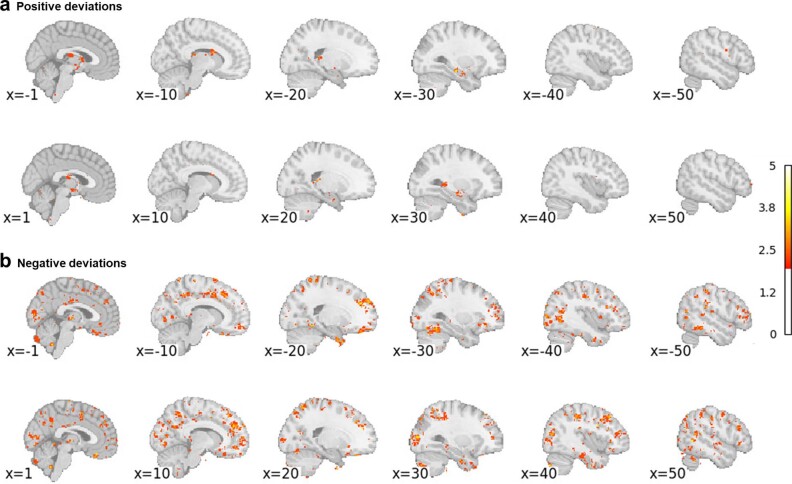
Spatial representation of the individual deviations. Percentage of deviations (positive deviations (**a**) and negative deviations (**b**)) from the normative model at each brain locus.

Next, we aimed to characterize individual neurobiological variability on top of the environmental signature, that is, the residuals of the normative model. To achieve this, we analyzed deviations from trajectories of the normative model by computing subject- and voxel-specific *z* scores reflecting more volume contractions (*z* < −2.6, negative deviations) or expansions (*z* > 2.6, positive deviations) relative to what was predicted by the model. Individual variability was highly stable across development (Extended Data Fig. 7), in line with the findings observed for the normative model. Negative deviations—that is, more volume contractions than predicted by the model—were widely prevalent in the brain, whereas positive deviations—that is, more volume expansions than predicted—were more focal, in the brain stem, thalamus and limbic system (Extended Data Fig. 8).

Finally, we calculated linear mixed models to determine whether this individual variability predicted subsequent psychopathological symptoms. Individual negative voxel-wise deviations at T1 predicted current and later anxiety, which was also the case when deviations at T2 were included in the model (Fig. 1c). No relation to other symptoms or with regard to positive deviations emerged (*P* > 0.05) and the model remained significant when controlled for other depressive symptoms, aggression and attention problems (*β* coefficient = 0.03, s.e. = 0.008, *P* = 0.0003) and all adversities (*β* coefficient = 0.06, s.e. = 0.01, *P* = 0.0001, *η*^2^ = 0.07).

## Discussion

Using a machine-learning approach to assess structural brain changes at the voxel level, we revealed compelling evidence for a neurobiological signature of a lifespan adversity exposure profile in the adult brain; this signature was stable over an 8-year period and could be replicated in an independent sample. In addition, we showed neuroanatomically distinct trajectories of brain expansions and contractions related to specific adversities, pointing to region-specific accelerated or delayed development. Moreover, we demonstrate that widespread individual-level deviations from the normative model are predictive of anxiety, indicating that interindividual differences in the expression of the adversity signature we report are clinically meaningful.

The design of our study enabled us to investigate the independent and combined long-term effects of adversities. Previous studies have predominantly focused on predicting neurobiological outcomes of adverse experiences within specific contexts, such as abuse or low socioeconomic status (for example, refs. ^5,6,36^). While this approach is valuable, for example, for identifying risk-sensitive periods, especially in early development, the correlation between adversities makes it difficult to establish the long-term effects of a specific adversity^2,4,37,38^. For instance, poverty—a deprivation-related adversity—is associated with various risks and negative outcomes at multiple levels, including individual-level issues such as substance abuse, family-level problems such as maltreatment and neighborhood poverty. Poverty can thus result in an accumulation of adversities over time. Therefore, it is essential to assess all environmental exposures throughout the lifespan to reveal how the adult brain may have adapted to adversity. Research indicating stronger predictions by accumulated adverse events than by single adversities with regard to adult psychopathology^37^ and telomere length^39^ supports the idea of a comprehensive assessment of exposures.

Our model suggests a persistent trace of adversities in the brain (Fig. 1b). By including different kinds of adversities such as obstetric complications, which are typically not considered in studies on early life adversities and brain alterations despite their link to cognitive^40^ and brain development^30,41^, we provide insight into how a specific type of adversity relates to changes in a particular brain region. This was, for example, revealed in the vmOFC (Extended Data Fig. 4). In typical brain development, increasing age is related to volume contractions in this region (Fig. 4). Of note, our findings reveal that diverse trajectories are associated with the following distinct adversity profiles: vmOFC volume contractions are observed after increased psychosocial adversities, and vmOFC volume expansions are observed after obstetric risks (likely involving preterm birth; Supplementary Fig. 2). While the former supports an accelerated vmOFC development, in line with previous findings^42^, the latter suggests a delayed maturation possibly due to deferred programmed cell death or decreased synaptic pruning. Notably, this is in line with previous reports on preterm birth as being related to volume increases regardless of developmental stage in the vmOFC^29,30,41,43–47^. Similar contrasting effects were observed in limbic regions such as the amygdala and hippocampus. In typical development, age-related expansions are noted. In the presence of psychosocial adversities (for example, Supplementary Tables 16, 18 and 20), age-related expansions exhibit a more pronounced pattern, while following obstetric risks, volume contractions are more pronounced (Supplementary Table 8). In conclusion, our findings suggest that psychosocial adversities may accelerate development in these limbic regions, while exposure to obstetric risks may delay maturation.

Our findings reveal robust structural brain changes linked to adversity that are stable in adulthood, consistent with earlier findings on persistent adversity-related alterations in functional circuitries^24,26^. It remains to be investigated how this adversity-related neuroplasticity offers potential for targeted interventions and whether it may be protective under certain environmental conditions. There is evidence that volumetric changes after exposure to severe deprivation in early childhood in the Romanian orphanages in the 1980s are manifest in the adult brain despite subsequent adoption into nurturing environments^23^. While the extreme adversity of the latter might not be directly applicable to the less severe exposure profile in this study, the stable neurobiological pattern observed in this study may potentially also entail reduced neural adaptability in adulthood. Further research is warranted to explore this, because (neural) adaptability may constitute a protective factor^48^.

Although there is exhaustive literature linking adversity to internalizing and externalizing psychopathology^37^, we show that negative deviations from the normative pattern (that is, more volume contractions) during young adulthood are predictive of current and later anxiety. Albeit we did not specifically predict the association with anxiety, findings of a recent meta-analysis may provide a possible explanation by showing that moderate adversities, such as those investigated here, promote heightened cortisol reactivity^49^. As a consequence, sensitivity to contextual cues is increased which might thus promote the maturation of brain regions that support increased vigilance. This, in turn, may manifest in more anxiety symptoms and hyperactivity of the stress system, exerting neurotoxic effects that are reflected in negative deviations.

## Limitations

Despite the longitudinal design of the MARS cohort, imaging data were obtained only in adulthood. The collection of neuroimaging data from individuals across all age groups—from infancy to old age—will be critical for investigating whether the timing of adverse experiences, especially during critical periods of brain development, can have differential effects on brain structure. Also, this will enable testing for cause–effect relationships. It is assumed that volume changes as observed in this study emerged due to adversities, yet this relationship might also turn out to be bidirectional or even the other way around^50,51^.

**Extended Data Fig. 9:**
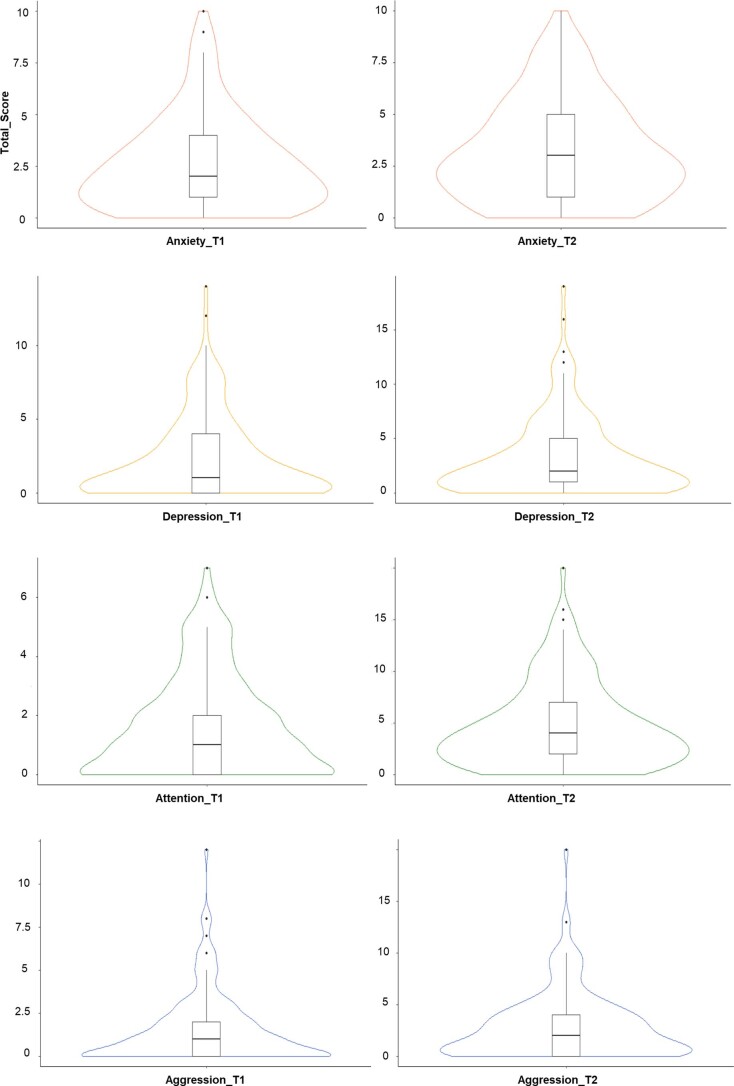
Psychopathology distribution in the MARS. The plots depict the distribution of anxiety (T1: range: 0-10, median: 2; T2: range: 0-10, median: 3), depression (T1: range: 0-14, median: 1, T2: range: 0-19, median: 2), attention (T1: range: 0-7, median: 1, T2: range: 0-20, median: 4) and aggression (T1: range: 0-12, median: 1, T2: range: 0-20, median: 2) at both time points (T1: n = 169, T2: n = 114). Box limits indicate the range of the central 50% of the data, with a central line marking the median value and whiskers extending a maximum of 1.5 times the Interquartile Range from the upper (75%) and the lower (25%) quartiles. Outliers beyond the whiskers are represented as individual data points.

In addition, the results warrant further validation in several respects. First, in particular, the structure coefficients (reflecting the contribution of each adversity to the overall brain associations) are sensitive to the cohort under investigation. This can be addressed by replicating these findings in cohorts with larger sample sizes, which would allow for improved normative modeling and more accurate predictions of adversity-specific effects. Second, the association between individual-based deviations and anxiety warrants replication in datasets with similar assessments to the MARS cohort. Longitudinal datasets acquired using the same scanner would be especially valuable as they would allow testing of how the normative model changes between time points. This was not possible in the MARS datasets due to the confounding effect of different scanning systems used at each time point. Third, given that the MARS sample predominantly comprised individuals without clinical psychopathology (Extended Data Fig. 9), it is important to conduct additional validation studies involving clinical populations to establish brain–psychopathology connections. Fourth, different behavioral and neurobiological correlates of adversity perception and acquisition have recently been discussed, which may influence the interpretation of our findings. More specifically, previous reports showed a stronger relationship between psychopathology and the subjective experience of adversity compared to the objective presence of adversity^52^ and showed that prospective records are more predictive of brain structure when compared to retrospective reports^22^. Although our adversity assessments did not allow for exploring these exact differences, we showed that prospectively acquired life events had a greater impact on brain morphometry compared to retrospectively collected trauma. However, it remains unclear whether these results are due to the nature of the report or the inherent nature of the adversities themselves. To address these questions, it could be interesting to apply the normative modeling approach presented here to such adversity assessment in future studies. Fifth, our instruments did not allow a distinction between threat and deprivation as dimensions of adversity, which have been shown to potentially accelerate and delay brain maturation in youth, respectively^16,53–56^. In general, our results do not point to differential correlates and instead suggest an acceleration of development, particularly in limbic structures, regardless of which of the psychosocial risks is considered. We speculate that differential effects of threat and deprivation may primarily occur in childhood and adolescence and may be less evident in the adult brain as adversities tend to be correlated and accumulate over time. This can be addressed by future studies that include repeated harmonized multilevel assessments allowing differentiation of facets and assessments of adversity, including threat and deprivation^57^, objective versus subjective and prospective and retrospective reports as well as the intensity, controllability and duration of the stressor and the contextual environment in which it occurs. Such an assessment of the so-called ‘eco-exposome’^4^ would require the integration of multimodal data, including e-diaries, sensors and also other biological features such as proinflammatory signaling^58^, and dysregulation of the hypothalamic–pituitary–adrenal axis^49,59^, which have been shown to be susceptible to adversity exposure.

To summarize, we show that adversities spanning from the prenatal period up to adulthood are associated with a persistent neural signature that is widespread in the brain and is stable during young adulthood. The direction of these adversity-related brain changes is region-, adversity- and timing-specific, which might be informative for translating these findings to therapies and efforts to improve public mental health. Of note, individual heterogeneity as reflected by volume contractions outside the normative range could predict current and future anxiety symptoms.

## Methods

The study was approved by the ethics committee of the University of Heidelberg (both for MARS and IMAGEN). Written informed consent was obtained from all participants, and they received monetary compensation for their involvement. Ethical approval for the public data was provided by the relevant local research authorities for the studies contributing data. For full details, see the main study publications given in the Supplementary Information.

### Study design

This investigation was conducted in the framework of the MARS, an ongoing cohort study of the long-term outcome of early risk factors^40^. Depending on pregnancy and birth history and on family background, infants were assigned to 1 of 9 groups of a two-factorial design with the degree of biological risk (obstetric complications) and the degree of psychosocial risk (no, moderate or high; for more information, see Supplementary Methods, purposive sampling strategy). Of 309 participants (80% of the original sample) participating in the 25-year assessment, a subsample took part in the neuroimaging session (*n* = 200, T1). After exclusion due to left-handedness or somatic diseases (*n* = 19), technical artifacts in the scans (*n* = 2) and missing data (*n* = 10), 169 healthy participants were included (58% females). A total of 118 individuals were rescanned at age 33 years/34 years (T2), but four had to be excluded due to technical artifacts. Twelve of those received a diagnosis at T2 (seven anxiety disorder, four major depression disorder and one substance use disorder). No statistical methods were used to predetermine sample sizes, but our sample sizes are similar to those reported in previous publications with a Gaussian model^32^. Data collection and analysis were not performed blind to the risk group of the participants.

### Assessments

#### Adversities

In the MARS sample, lifetime adversities encompassed maternal smoking during pregnancy (standardized interview with the mother conducted at the 3-month assessment; nonsmokers, 1–5 cigarettes per day, >5 cigarettes per day), prenatal maternal stress (standardized parent interview was conducted at the 3-month assessment concerning worries, mood problems and positive experiences during pregnancy), maternal sensitivity (videotapes of a 10-min standardized nursing and play situation between mothers and their 3-month olds), obstetric adversity (assessed at the age of 3 months, adverse conditions during pregnancy, delivery and postnatal period such as preterm labor, asphyxia or seizures; Supplementary Fig. 2), psychosocial family adversity (standardized interview assessed at 5 time points between 3 months of age and 11 years, adverse characteristics of the parents (low educational level, broken home history or delinquency, poor coping skills, psychopathology), their partnership (early parenthood, one-parent family, unwanted pregnancy, marital discord) and the family environment (overcrowding, poor social integration and support, severe chronic life difficulties))^60^, childhood trauma (at the age of 23 years, Childhood Trauma Questionnaire^61^), life events (from 3 months to 15 years with semi-structured parent interview, afterwards interview with participants^62^, burdensome life events in the past year encompassing the family, school, parents, health, legal troubles and living conditions). Detailed descriptions can be found in the Supplementary Methods and the MARS assessments along with the correlative structure of the adversities in Extended Data Fig. 1b.

#### Adult psychopathology

The Young Adult Self-Report^63^ and the Adult Self-Report^64^ were used to measure clinical symptoms on the basis of Diagnostic and Statistical Manual of Mental Disorders (DSM-IV) criteria at the ages of 25 years and 33 years, respectively. Based on our own previous reports on adversity effects on psychopathology^21,38,65^ and those by others (for example, ref. ^1^), we focused on the raw scores of specific subscales reflecting the internalizing and the externalizing spectrum, that is, anxiety, depression, aggression and attention-deficit/hyperactivity disorder.

### Anatomical images

At the 25-year assessment, we acquired 1 × 1 × 1 mm T1-weighted anatomical images with 192 slices covering the whole brain using a 3T scanner (Magnetom TRIO, Siemens) with a standard 12-channel head coil. At the 33-year-assessment high-resolution anatomical images with 208 slices covering the whole brain were acquired using a 3T scanner (PrismaFit, Siemens) with a 32-channel head coil.

### Anatomical data preprocessing

Preprocessing for both datasets was done using the anatomical processing tools implemented in FSL (FMRIB Software Library, v6.0.5, details described in the Supplementary Methods). For further analyses, affine and log-transformed JDs of the deformation fields were used as features (Supplementary Methods).

### Statistical analysis

#### Adversity scores

All adversity scores were categorized to yield a maximum of four bins with a minimum of ten individuals in one bin (Supplementary Fig. 1). However, results were similar when scores were not binned (Extended Data Fig. 3c).

#### Normative models

We estimated normative models of morphometric variation (quantified by the JDs of the deformation field from a nonlinear image registration) for (1) adversity and (2) normative brain development and aging across the lifespan. For all models, a Bayesian linear regression (BLR) model was applied using the Predictive Clinical Neuroscience toolkit (PCNtoolkit) software (https://pcntoolkit.readthedocs.io/en/latest). For the adversity models, we included all developmental risks, TIV and sex as covariates and used a linear BLR model with Gaussian noise. For the developmental normative models, we used age, sex and scanning site as covariates with a B-spline basis expansion over age along with likelihood warping to model non-Gaussianity in line with prior study^66^. Further details are provided in the Supplementary Methods. For the adversity-related normative model, the whole sample served as a reference because the MARS is a cohort study consisting of individuals with lifetime adversity, of which only a subset will go on to develop psychopathology. Predictions were derived in an unbiased manner under tenfold cross-validation for the adversity model and using a split-half holdout sample for the aging model. Briefly, this Bayesian approach calculates the probability distribution over linear coefficients defining functions that fit the data while specifying a prior over all possible coefficient values and updating these distributions based on evidence (that is, observed data). As such, it yields unbiased estimates of generalizability and inferences with increasing uncertainty with fewer data. While this increases the conservativeness of this approach and renders deviations harder to detect in regions with fewer data points, the deviation statistics ought to be interpreted with respect to this specific cohort.

**Extended Data Fig. 10:**
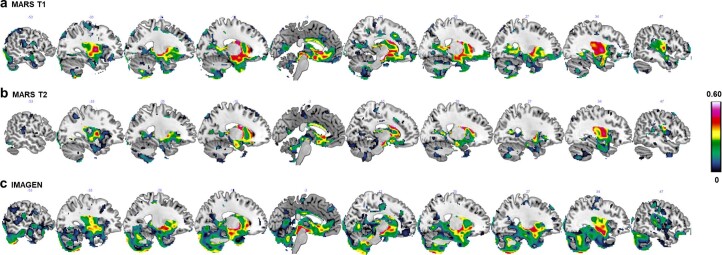
Spatial distribution of explained variance. Normative model based lifetime adversity, sex and TIV in **a**, the MARS sample at T1, **b**, the MARS sample at T2 and **c**, the IMAGEN sample.

The accuracy of the normative model showing the long-term adversity signature was evaluated using the correlation between the true and the predicted voxel values (ρ) and the standardized mean squared error (Supplementary Fig. 3), which is in accordance with previous studies^19^. In general, the models explained up to 59% of the variance in JD alterations (see Extended Data Fig. 10 for the adversity and Fig. 4 for the aging model). Structure coefficients, which reflect the correlation between a predictor and the expected outcome, were calculated to assess the contribution of each single adversity to the predicted model.

To estimate a pattern of regional deviations from typical brain structure for each participant, we derived normative probability maps (NPM) that quantify the voxel-wise deviation from the normative model. This was done by calculating an individual-specific *z* score^27^ indicating the difference between the prediction at each brain location and true brain structure scaled by the prediction variance.

The NPMs were thresholded at *z* = ±2.6 (that is, *P* < 0.005) as in refs. ^32,67,68^ to facilitate the comparison across participants and to have a more sensitive marker for small individual deviations when compared to false discovery rate correction. Before testing the association with psychopathology, individual deviations were subjected to a Box–Cox transformation to normalize the data.

#### Multivariate models

In light of our small sample size and the number of adversities, a principal component analysis with varimax rotation was performed using sklearn (0.24.2) implemented in Python 3.6. A model was estimated to predict brain changes based on adversities, using three PCs that explained 63% of the variance (see Supplementary Methods for further details). Estimated predictions were based on four (random) sampling points of the loadings and scaled by the square root of the eigenvalue.

#### Relation to psychopathology

Linear mixed models with random intercepts for all the level-1 predictors (deviations and time) were fitted. Model 1 tested the prediction of psychopathology (distribution shown in Extended Data Fig. 9) assessed at T1 and T2 based on deviations acquired at T1. The model was corrected for four psychopathology outcomes and two deviation scores (positive/negative), yielding a corrected *P* value of 0.008 (two-sided test) as being significant. Model 2 tested the replication of this analysis taking additionally the deviations at T2 into account. All models were designed with R-4.1.0 packages lme4 (v1.1.27.1)^69^, and lmerTest (v3.1.3)^70^ and visualized with sjPlot (v2.8.12)^71^. Only dimensional psychopathology was tested, given the low number of diagnoses at T2.

#### Sensitivity analysis

Several sensitivity analyses were conducted which confirmed the robustness of the results (Supplementary Methods).

### Replication sample

The Mannheim subsample (*n* = 115) of the IMAGEN consortium had a similar age (22 years), sex distribution (56% females) and scanning parameters. Two adversity measures (negative life events^72^ and childhood trauma^61^) were available in the IMAGEN sample, which allowed testing for replication of the neurobiological signature of adversity (details in the Supplementary Methods).

### Reporting summary

Further information on research design is available in the Nature Portfolio Reporting Summary linked to this article.

## Online content

Any methods, additional references, Nature Portfolio reporting summaries, source data, extended data, supplementary information, acknowledgements, peer review information; details of author contributions and competing interests; and statements of data and code availability are available at 10.1038/s41593-023-01410-8.

## Supplementary information

Supplementary Information Supplementary Figs. 1–4, Supplementary Methods and Supplementary Tables 1–30.

Reporting Summary

## Data Availability

Data supporting the findings of this study (that is, the MARS and IMAGEN samples) are available upon reasonable request from the corresponding authors, subject to local ethics committee requirements. Publicly available data derived from the lifespan normative models are available via the repositories contributing the data (Cam-CAN: https://www.cam-can.org/index.php?content=dataset; PNC: https://www.nitrc.org/projects/pnc; UKB: https://www.ukbiobank.ac.uk; OASIS: https://www.oasis-brains.org; HCP: https://www.humanconnectome.org/study/hcp-young-adult). Source data are provided with this paper.

## Source data

Source Data Fig. 1 Voxel-wise Pearson correlations (two-sided) between the true morphometric changes of the JDs and the predicted values in the normative models built on adversities, sex and TIV for MARS T1, MARS T1 (only intersection sample T1/T2), MARS T2, IMAGEN.
Source Data Fig. 2 Top 2% of the structure coefficients for MARS T1 and MARS T2 (*_t2.txt). For each adversity, the top 2% of the positive (top2) voxel-wise correlations and the top 2% of the negative (low2) voxel-wise correlations (negative multiplied by −1) between adversity and the predicted morphometric changes of the JDs of deformation fields for T1 and T2 (*_t2.txt) are in the tables. Family_adv, family adversity; life_events, stressful life events; mat_sens, maternal sensitivity; smoke_preg, maternal smoking during pregnancy.
Source Data Fig. 3 Voxel-wise slope values of the linear regression predicting JD development based on the first PC (PC1), the second (PC2) and the third (PC3).
Source Data Fig. 4 Voxel-wise explained variance by the age-related normative model.

## Code availability

Code is available at https://github.com/amarquand/PCNtoolkit.
