# Supplementary material for: A stable and replicable neural signature of lifespan adversity in the adult brain
Source: Nat Neurosci. 2023 Aug 21;26(9):1603–12. doi: 10.1038/s41593-023-01410-8 (PMC10471497; doi:10.1038/s41593-023-01410-8)
Supplement: Supplementary file 1 — Supplementary Figs. 1–4, Supplementary Methods and Supplementary Tables 1–30. [file 41593_2023_1410_MOESM1_ESM.pdf]

nature neuroscience

Article https://doi.org/10.1038/s41593-023-01410-8

A stable and replicable neural signature of lifespan adversity in the adult brain

In the format provided by the authors and unedited

SUPPLEMENTARY INFORMATION FILE

SUPPLEMENTAL FIGURES

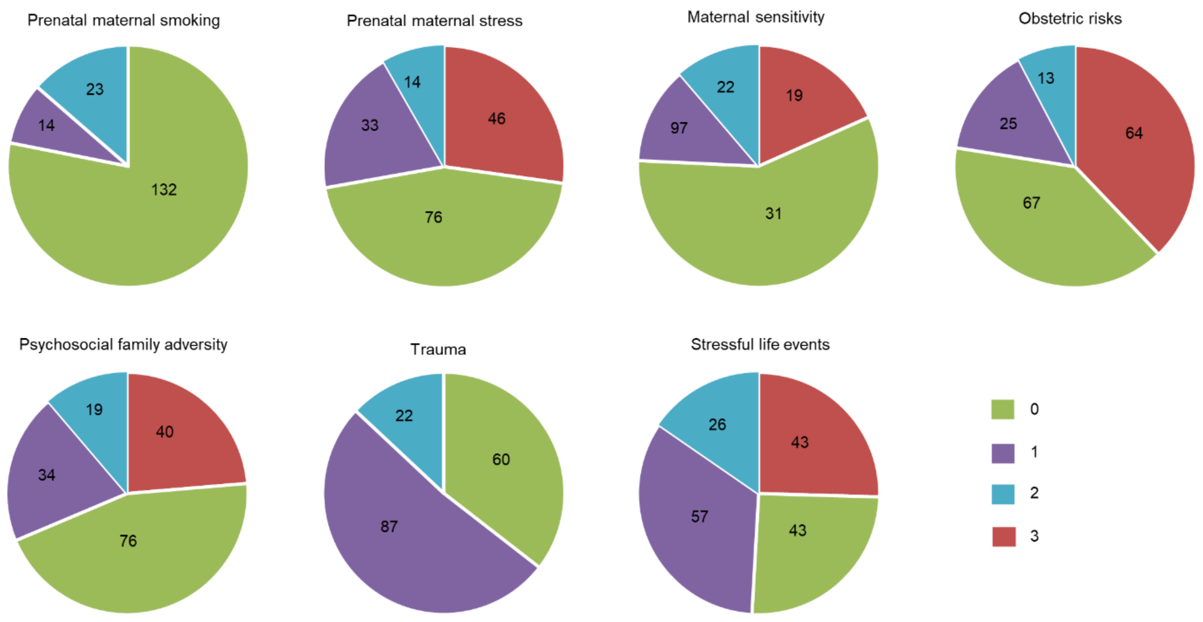

Supplemental Figure 1. Adversity bins. Pie charts including the number of participants for each bin of each category. 0= no adversity, 1=low adversity, 2= moderate adversity, 3= high adversity.

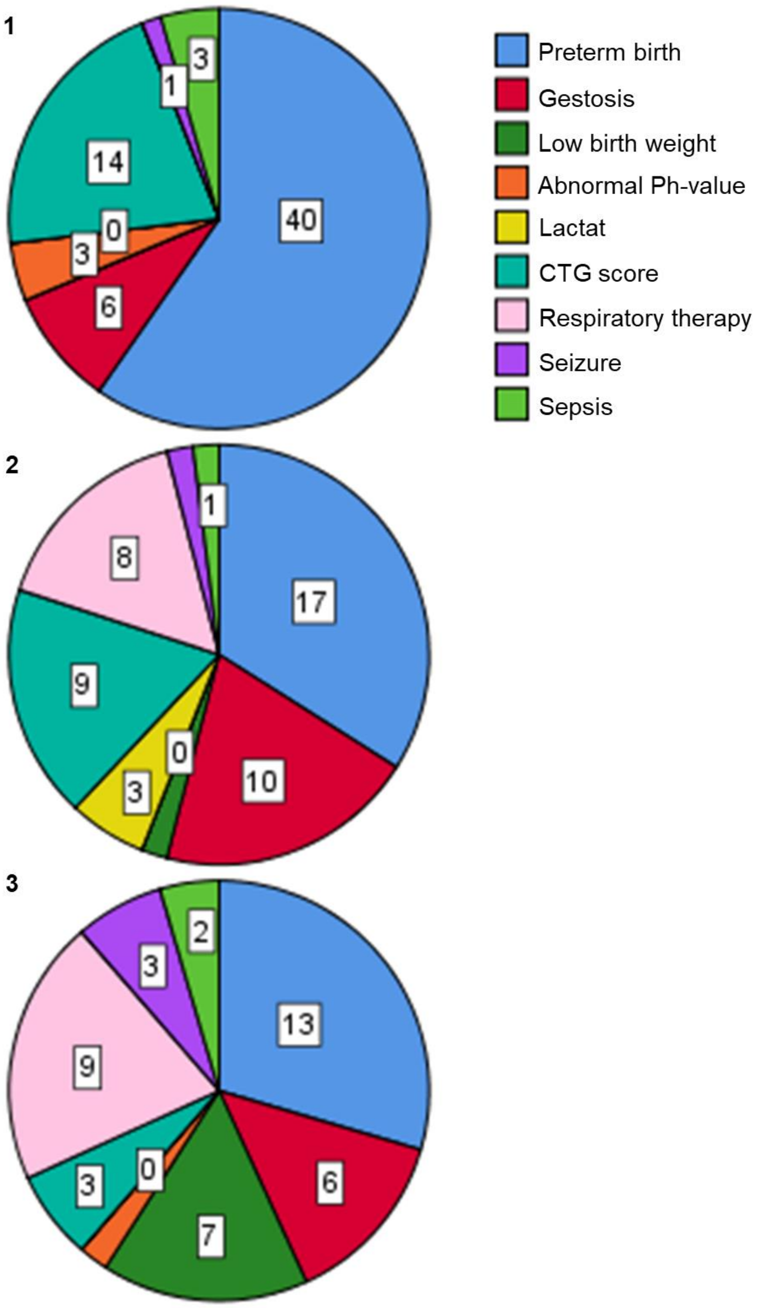

Supplemental Figure 2. Obstetric risk. Distribution of obstetric risk factors across the three exposure bins.

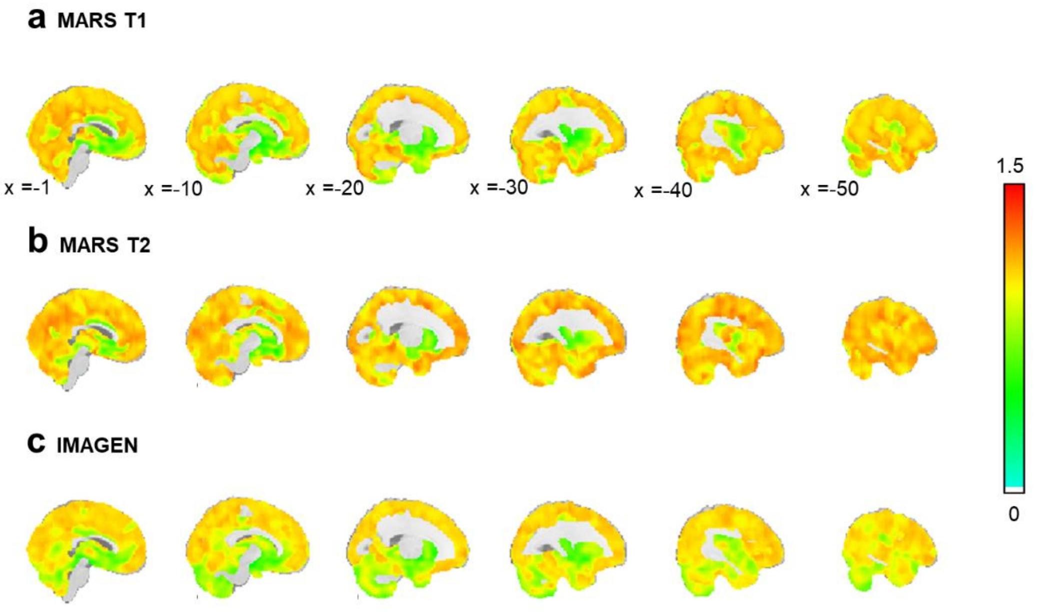

Supplemental Figure 3. Model accuracy. The figure shows the standardized mean squared error of true and predicted mean of morphometric changes as a function of adversity in a, the MARS sample at T1, b, the MARS sample at T2 and c, the IMAGEN sample. Model performance was better in subcortical than in cortical regions.

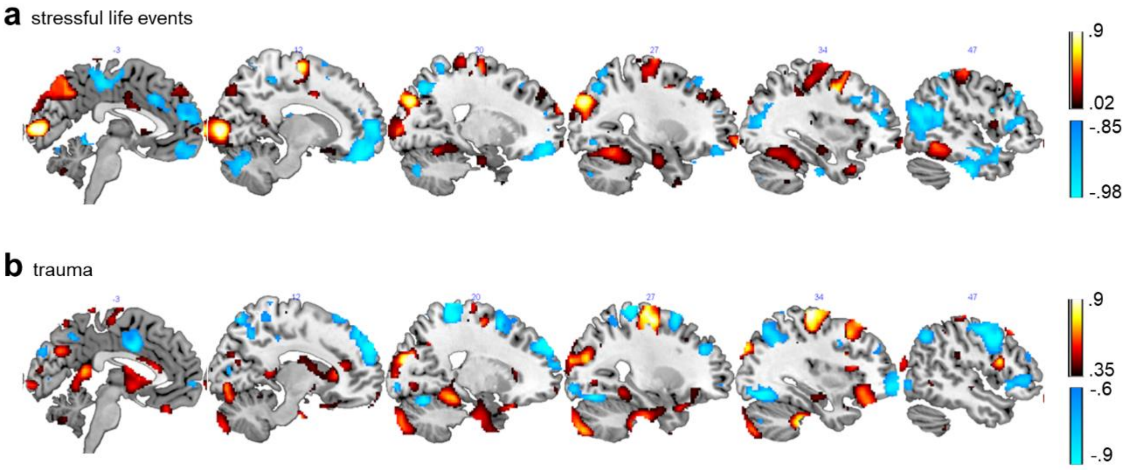

Supplemental Figure 4. IMAGEN structure coefficient. Spatial representation of the top 2% of the voxel-wise contribution of each adversity on predicted morphometric changes identified based on structure coefficients in the IMAGEN sample. a, stressful life events and b, trauma.

ONLINE METHODS

Study Design MARS

All groups had about equal size, with a slight oversampling in the high-risk combinations and with sex evenly distributed in all subgroups. A total of 384 infants born between February 1, 1986, and February 28, 1988, were recruited from 2 obstetric and 6 children's hospitals of the Rhine-Neckar region of Germany. To control confounding effects of family environment and infant medical status, only firstborn singletons of German speaking parents with no severe physical handicaps, obvious genetic defects, or metabolic diseases were selected. Participation rate at the time of recruitment was 64.5%, with a slightly lower rate in parents from psychosocially disadvantaged backgrounds. All families were Caucasians.

MARS Assessments

Maternal smoking during pregnancy. Was determined by a standardized interview with the mother conducted at the 3-month assessment and classified as nonsmokers, smoking 1-5 cigarettes per day (cig./d) and more than 5 cigarettes per day for further details see [1].

Prenatal maternal stress. A standardized parent interview was conducted at the 3-month assessment. 11 questions were asked concerning worries, mood problems, as well as positive experiences during pregnancy. Mothers were requested to judge separately for the first and the second/third trimesters. As associations of prenatal stress in mid- and late pregnancy with behavioral outcome in the offspring have been reported to be largest [2], only prenatal stress during the second and third trimester was included.

Early mother-child interaction. As described in Holz et al.[3], videotapes of a 10-min standardized nursing and play situation between mothers and their three-month-olds at our lab were recorded and evaluated by trained raters (κ>0.83) using a modified version of the category system for micro-analysis of the early mother–child interaction [4, 5]. Raters were blind to parental and child risk status. Nine measures of mother–infant interaction behavior were formed by coding a behavior as present or absent in a total of 120 five-second intervals. Maternal stimulation included all attempts to attract the infant´s attention or to establish contact with him/her (vocal, facial or motor) and was coded when the baby was gazing at the mother or when the behaviors were clearly directed at the child. The scores were z-transformed and recoded such that higher scores represent lower stimulation.

Obstetric adversity. At the age of 3 months, an obstetric adversity score was obtained by counting the presence of 9 adverse conditions during pregnancy, delivery, and postnatal period such as preterm labor, asphyxia, or seizures. See Figure S2 for its composition. This measure of adversity was included due to the enrichment of the MARS sample with respect to obstetric risks and their well-known effects on socio-cognitive[6] and neural development[7-12].

Psychosocial adversity. Information on adverse characteristics of the parents (low educational level, broken home history or delinquency, poor coping skills, psychopathology), their partnership (early parenthood, one-parent family, unwanted pregnancy, marital discord) and the family environment (overcrowding, poor social integration and support, severe chronic life difficulties) was assessed according to an 'enriched' family adversity index [13] by a standardized parent interview conducted at each assessment (n=5) until the age of 11 years (range 0-9, M=2.95; SD=2.05). The score is created such that events that reflect only one possible exposure during lifetime (e.g. unwanted pregnancy) are also only counted once.

Childhood trauma. At the age of 23, participants completed the brief screening version of the Childhood Trauma Questionnaire (CTQ)[14]. The CTQ entails a retrospective assessment of five types of self-reported childhood maltreatment, i.e. sexual, physical, and emotional abuse, and emotional and physical neglect. The scores of all subscales were summed up.

Life events. To assess exposure to life stress (LS) across the life span, a semi-structured parent interview was conducted until the age of 15 years. The young adults were interviewed from the age of 19 years onwards. The interview, which was a modified and shortened version of the Munich Events List [15], evaluated the occurrence of adverse life events during a period of one year prior to the assessment. The items covered all relevant areas of children's and young adults' LS, including family, school, parents, health, legal troubles, and living conditions, such as birth of a sibling, death of a close relative or parents` separation for which the participant indicated a subjective burden. A composite score was computed by summing up the z-standardized scores from the ten assessments between the age of 3 months and 25 years.

Anatomical image preprocessing.

T1-weighted anatomical images with 192 slices covering the whole brain were acquired at the 25-year assessment using a 3T scanner (matrix 256x256, repetition time=2300ms, echo time=3.03ms, 50% distance factor, field of view 256x256x192mm, flip angle 9°). Preprocessing of the anatomical images entailed the following steps. First, images were reoriented to the standard (MNI) orientation [fslreorient2std], automatically cropped [robustfov] and bias-field corrected (RF/B1-inhomogeneity-correction) [FAST]. Then registered to standard space (linear and non-linear) [FLIRT and FNIRT], followed by brain-extraction [FNIRT-based or BET] as well as tissue-type segmentation [FAST] and subcortical structure segmentation [FIRST]. Data were visually inspected and evaluated by an experienced rater (NH).

As done previously[16], we selected the nonlinear Jacobian determinants as feature for the normative model given that they quantify the overall degree of tissue expansion or contraction required to match each image to the population template. Thereby, they contain all information necessary to warp the subject to template and avoid making a sometimes arbitrary distinction between what can be considered white and grey matter, and is particularly valuable in avoiding partial volume effects. Notably, these features have been shown have a stronger relationship with demographic variables than more commonly used features (e.g. modulated grey matter density[17]). The images were affine and log transformed and masked by a grey matter template.

In addition, we estimated the normative model on grey matter density, which we calculated by warping grey matter to MNI space and subsequent multiplication by the jacobian determinants and smoothing with a 6mm kernel.

Normative Models based on adversity

Bayesian Linear Regression was chosen based on our own reports demonstrating linear relationships between adversities and brain structure and others reporting long-term effects with respect to brain morphometry[18]. We estimated the hyper-parameters of this model (namely prior weight precision and noise precision) using an Empirical Bayes approach as we have done in prior work[19-21]. We evaluated the performance of our model by making out of sample predictions under 10-fold cross validation.

Individual-specific *Z* score[22] indicated the difference between the prediction (mean, $\hat{y}_{ij}$) at for each subject (i) at each brain location (j) and true brain structure (y_{ij}) scaled by the prediction

variance [expected level of variation σ^2_{ij} and variance learned from the normative distribution (σ^2_{nj})]:

$$z_{ij} = \frac{y_{ij} - \hat{y}_{ij}}{\sqrt{\sigma^2_{ij} - \sigma^2_{nj}}}$$

Normative modeling was run using python 3.6 and the PCNtoolkit package (version 0.19).

Normative Models based on age

Data from 9 sites were combined to create the initial full sample. These sites are described in detail in supplementary tables 25 and 26 including the sample size, age (mean and standard deviation), and sex distribution of each site. In brief, these were derived by including data from the following publicly available sources: The Philadelphia Neurodevelopmental Cohort (PNC)[23], Cam-CAN[24], Human Connectome Project[25], UK Biobank[26, 27] and OASIS3[28] in addition to the MARS and IMAGEN data all processed using an identical pipeline. This is a subset of the data reported in our previous work[21] and we followed the same procedures reported in that manuscript with regard to quality control.

Normative modeling was run using python 3.8 and the PCNtoolkit package (version 0.26). Bayesian Linear Regression (BLR) with likelihood warping (‘sinarcsinsh’ warping function)[20, 21] was used to model voxel-wise JD development from a vector of covariates (age, sex, and site). For each voxel y is predicted as:

$y=w^T\phi(x)+\epsilon$

where w^T is the estimated weight vector, $\phi(x)$ is a basis expansion of the of covariate vector x, consisting of a B-spline basis expansion (cubic spline with five evenly spaced knots) to model non-linear effects of age, and $\epsilon=N(0,\beta^{-1})$ a Gaussian noise distribution with mean zero and noise precision term β (the inverse variance).

Principal component analysis

The Kaiser–Meyer–Olkin measure of sampling adequacy was .69, representing a relatively good factor analysis, and Bartlett’s test of Sphericity was significant ($p < .001$), indicating that correlations between items were sufficiently large for performing a PCA. Only factors with eigenvalues ≥ 1 were considered[29, 30]. Examination of Kaiser’s criteria and the scree-plot yielded empirical justification for retaining three factors with eigenvalues exceeding 1 which accounted for 63% of the total variance. Most adversities loaded highly on only one of the two factors (see supplemental Table 22). The predictive models were estimated for each of the three PC´s separately, based on 4 (random) sampling points of the loadings, scaled by the square root of the eigenvalue and the respective adversity mean added.

Sensitivity analyses

First, to ensure that the results were not affected by the categorization of the adversity scores, two additional separate normative model were set up with the three principal components (not binned) and with the z-standardized (not binned) scores and, respectively. Second, to evaluate whether the stability of the patterns from T1 to T2 was not affected by the subject drop-out at T2, a further normative model at T1 was fitted including only the participants that had follow-up data. Third, to ensure that results are not affected by the inclusion of TIV, the whole analysis pipeline was repeated without this variable. Forth, to elaborate the effect of adversity

only, normative models excluding sex and total intracranial volume (TIV) were created in both samples. Fifth, we excluded obstetric adversities given its qualitatively different nature of risks. Sixth, we used modulated grey matter images as outcome feature instead of JDs. Seventh, given the recent discussion on a differential impact of adversity depending on the subjective appraisal, we additionally created a normative models based on retrospectively reported trauma (CTQ) and the prospectively reported life events with the constraint that both measures cover slightly different forms of adversity.

IMAGEN

Anatomical images. MRI was performed on a 3T scanner (Siemens Trio). High-resolution anatomical MR images were obtained using a standardized 3D T1-weighted magnetisation prepared rapid acquisition gradient echo (MPRAGE) sequence based on the ADNI protocol (http://adni.loni.usc.edu/ methods/mri-analysis/mri-acquisition/). The parameters were as follows: repetition time = 2300 ms, echo time = 2.93 ms, flip angle = 9˚, 1.1x1.1x1.1 mm voxel size.

Assessments. During all 4 assessment waves (14, 16, 19, 22 years), the participants completed the Life Events Questionnaire (LEQ)[31] that was adapted for their age. The participants indicated the experience of an event and how they felt after the event. Negative life events were coded once if they felt “unhappy” after the event and twice if they felt very unhappy (mean=18.26, SD=8.75, range=5-40). The sum scores were z-transformed to create a composite score.
The CTQ was assessed at the age of 19 years. The total score across all scales was used (mean=31.68., SD=7.64, range=25-73).

REFERENCES

1. Holz, N.E., *et al.* Effect of prenatal exposure to tobacco smoke on inhibitory control: neuroimaging results from a 25-year prospective study. *JAMA Psychiatry* **71**, 786-796 (2014).
2. Rice, F., *et al.* The links between prenatal stress and offspring development and psychopathology: disentangling environmental and inherited influences. *Psychological medicine* **40**, 335-345 (2010).
3. Holz, N.E., *et al.* Early maternal care may counteract familial liability for psychopathology in the reward circuitry. *Social cognitive and affective neuroscience* **13**, 1191-1201 (2018).
4. Jörg, M., *et al.* [Category system for microanalysis of early mother-child interaction]. *Z. Kinder. Jugendpsychiatr. Psychother.* **22,** , 97-106 (1994).
5. Schmid, B., *et al.* Quality of early mother-child interaction associated with depressive psychopathology in the offspring: a prospective study from infancy to adulthood. *J Psychiatr Res* **45**, 1387-1394 (2011).
6. Laucht, M., *et al.* Behavioral sequelae of perinatal insults and early family adversity at 8 years of age. *J Am Acad Child Adolesc Psychiatry* **39**, 1229-1237 (2000).
7. Nam, K.W., *et al.* Alterations in cortical thickness development in preterm-born individuals: Implications for high-order cognitive functions. *NeuroImage* **115**, 64-75 (2015).
8. Nosarti, C., *et al.* Preterm birth and structural brain alterations in early adulthood. *Neuroimage Clin* **6**, 180-191 (2014).
9. Nosarti, C., *et al.* Grey and white matter distribution in very preterm adolescents mediates neurodevelopmental outcome. *Brain* **131**, 205-217 (2008).
10. Zhou, L., *et al.* Brain gray and white matter abnormalities in preterm-born adolescents: A meta-analysis of voxel-based morphometry studies. *PLoS One* **13**, e0203498 (2018).
11. Sripada, K., *et al.* Trajectories of brain development in school-age children born preterm with very low birth weight. *Scientific reports* **8**, 15553 (2018).
12. Karolis, V.R., *et al.* Volumetric grey matter alterations in adolescents and adults born very preterm suggest accelerated brain maturation. *NeuroImage* **163**, 379-389 (2017).
13. Holz, N.E., *et al.* Ventral striatum and amygdala activity as convergence sites for early adversity and conduct disorder. *Soc Cogn Affect Neurosci* (2016).
14. Bernstein, D.P., *et al.* Development and validation of a brief screening version of the Childhood Trauma Questionnaire. *Child Abuse Negl* **27**, 169-190 (2003).
15. Maier-Diewald, W., Wittchen, H.-U., Hecht, H. & Werner-Eilert, K. *MEL - Münchner Ereignisliste* (Max Planck Institute of Psychiatry, Munich (DE), 1983).
16. Holz, N.E., *et al.* Age-related brain deviations and aggression. *Psychological medicine*, 1-10 (2022).
17. Monte-Rubio, G.C., Falcon, C., Pomarol-Clotet, E. & Ashburner, J. A comparison of various MRI feature types for characterizing whole brain anatomical differences using linear pattern recognition methods. *NeuroImage* **178**, 753-768 (2018).
18. Gehred, M.Z., *et al.* Long-term Neural Embedding of Childhood Adversity in a Population-Representative Birth Cohort Followed for 5 Decades. *Biol Psychiatry* **90**, 182-193 (2021).
19. Huertas, I., *et al.* A Bayesian spatial model for neuroimaging data based on biologically informed basis functions. *NeuroImage* **161**, 134-148 (2017).
20. Fraza, C.J., Dinga, R., Beckmann, C.F. & Marquand, A.F. Warped Bayesian linear regression for normative modelling of big data. *NeuroImage* **245**, 118715 (2021).
21. Rutherford, S., *et al.* Charting brain growth and aging at high spatial precision. *Elife* **11** (2022).

22. Marquand, A.F., Rezek, I., Buitelaar, J. & Beckmann, C.F. Understanding Heterogeneity in Clinical Cohorts Using Normative Models: Beyond Case-Control Studies. *Biological Psychiatry* **80**, 552-561 (2016).
23. Satterthwaite, T.D., *et al.* Neuroimaging of the Philadelphia neurodevelopmental cohort. *NeuroImage* **86**, 544-553 (2014).
24. Shafto, M.A., *et al.* The Cambridge Centre for Ageing and Neuroscience (Cam-CAN) study protocol: a cross-sectional, lifespan, multidisciplinary examination of healthy cognitive ageing. *BMC Neurol* **14**, 204 (2014).
25. Van Essen, D.C., *et al.* The WU-Minn Human Connectome Project: an overview. *NeuroImage* **80**, 62-79 (2013).
26. Sudlow, C., *et al.* UK biobank: an open access resource for identifying the causes of a wide range of complex diseases of middle and old age. *PLoS Med* **12**, e1001779 (2015).
27. Miller, K.L., *et al.* Multimodal population brain imaging in the UK Biobank prospective epidemiological study. *Nat Neurosci* **19**, 1523-1536 (2016).
28. Marcus, D.S., Fotenos, A.F., Csernansky, J.G., Morris, J.C. & Buckner, R.L. Open access series of imaging studies: longitudinal MRI data in nondemented and demented older adults. *Journal of cognitive neuroscience* **22**, 2677-2684 (2010).
29. Guttman, L. Some necessary conditions for common factor analysis. *Psychometrika* **19**, 149-161 (1954).
30. Kaiser, H.F. The application of electronic computers to factor analysis. *Educational and Psychological Measurement* **20**, 141-151 (1960).
31. Newcomb, M. A multidimensional assessment of stressful life events among adolescents: Derivation and correlates. *Journal of Health and Social Behavior* **22**, 400-415 (1981).

Tables

Table S1. Correlation between true and predicted JDs based on adversities, sex and TIV at T1.

Voxels	ρ	MAX X (mm)	MAX Y (mm)	MAX Z (mm)	Region
64594	0.804	12	-14	-16	Brainstem/Hippocampus
872	0.46	-32	54	30	Superior Frontal Gryus
384	0.434	42	-68	54	Lateral Occipital Gyrus
104	0.413	26	-46	46	Superior Parietal Lobule
65	0.2	-6	14	70	Superior Frontal Gyrus
41	0.361	36	38	10	Frontal Pole
32	0.471	-28	32	26	Middle Frontal Gyrus
19	0.309	10	-30	78	Precentral Gyrus
17	0.372	36	-98	-2	Inferior Occipital Gyrus
17	0.158	-54	-38	-10	Middle Temporal Gyrus
17	0.21	2	-14	66	Precentral Gyrus
17	0.398	34	10	28	Precentral Gyrus
15	0.153	14	58	38	Frontal Pole
15	0.241	-20	16	68	Superior Frontal Gyrus
12	0.163	-42	-6	62	Precentral Gyrus.
11	0.158	-56	-10	46	Postcentral Gyrus.
10	0.189	42	-52	54	Superior Parietal Lobule.

Note: ρ=correlation coefficient

Table S2. Correlation between true and predicted JDs based on adversities, sex and TIV at T1 in limbic regions.

Region of Interest	hemisphere	Voxels	ρ	MAX X (mm)	MAX Y (mm)	MAX Z (mm)
amygdala	l	595	0.71	-12	-10	-14
	r	694	0.794	12	-10	-14
hippocampus	l	1045	0.76	-10	-12	-18
	r	1047	0.8	12	-12	-16
medial frontal gyrus		1354	0.684	-8	30	-12
anterior cingulate gyrus		2450	0.648	-4	32	-12

Note: ρ=correlation coefficient, unilateral masks used for the amygdala and the hippocampus, bilateral masks used for the medial frontal gyrus and the anterior cingulate gyrus

Table S3. Correlation between true and predicted JDs based on adversities, sex and TIV at T2.

Voxels	ρ	MAX X (mm)	MAX Y (mm)	MAX Z (mm)	Region
45161	0.768	8	-6	6	Thalamus
1189	0.393	-8	28	56	Superior Frontal Gyrus
509	0.388	-24	-82	48	Precuneus
388	0.421	42	14	56	Middle Frontal Gyrus
249	0.327	-34	-54	48	Inferior Parietal Lobule
202	0.515	14	14	40	Cingulate Gyrus
184	0.211	-20	-52	66	Superior Parietal Lobule
165	0.272	-8	62	-8	Medial Frontal Gyrus
144	0.239	34	-80	40	Precuneus
133	0.278	-18	-62	30	Occipital Lobe
126	0.388	28	-44	40	Parietal Lobe
119	0.206	50	-22	58	Postcentral Gyrus
108	0.228	14	-60	40	Precuneus
108	0.256	-52	-14	48	Postcentral Gyrus
90	0.222	6	60	24	Superior Frontal Gyrus
87	0.509	-22	-4	48	Middle Frontal Gyrus
67	0.239	-46	34	36	Middle Frontal Gyrus.
52	0.189	-52	-14	-22	Middle Temporal Gyrus
50	0.393	10	-104	12	Cuneus
41	0.156	-44	4	46	Middle Frontal Gyrus
40	0.278	-36	-28	68	Postcentral Gyrus

35	0.234	-10	68	24	Frontal Pole
32	0.162	16	44	40	Superior Frontal Gyrus
28	0.162	62	-48	44	Inferior Parietal Lobule
26	0.399	-46	40	-20	Middle Frontal Gyrus
25	0.184	-44	18	-14	Inferior Frontal Gyrus
24	0.189	-30	-4	56	Precentral Gyrus
23	0.145	-52	30	-4	Inferior Frontal Gyrus
23	0.184	50	-82	14	Middle Occipital Gyrus
22	0.355	-22	-28	52	Postcentral Gyrus
21	0.25	24	-40	-60	Cerebellum
20	0.134	-46	-64	26	Middle Temporal Gyrus
20	0.294	-18	-36	-42	Cerebellum
19	0.2	-50	-36	-8	Middle Temporal Gyrus
19	0.515	-24	30	28	Middle Frontal Gyrus
18	0.151	12	-90	18	Cuneus
17	0.167	-42	54	18	Superior Frontal Gyrus.
17	0.156	-28	-76	-22	Declive
11	0.189	40	-46	50	Inferior Parietal Lobule
10	0.129	30	38	32	Middle Frontal Gyrus
10	0.134	-28	-68	60	Lateral Occipital Cortex
10	0.129	16	14	60	Superior Frontal Gyrus.

Note: ρ=correlation coefficient

Table S4. Correlation between true and predicted JDs based on adversities, sex and TIV at T2 in limbic regions.

Region of Interest	hemisphere	Voxels	ρ	MAX X (mm)	MAX Y (mm)	MAX Z (mm)
amygdala	l	465	0.63	-12	-12	-16
	r	516	0.669	14	-12	-16
hippocampus	l	1045	0.76	-10	-12	-18
	r	594	0.686	14	-14	-16
	r	158	0.504	22	-42	0
medial frontal gyrus		428	0.636	-8	28	-14
		11	0.167	-6	50	-24
		10	0.14	-8	56	-10
anterior cingulate gyrus		990	0.619	6	10	24
		137	0.548	0	34	-12
		36	0.448	14	16	34
		20	0.222	14	-14	38
		3	0.129	4	30	0
		3	0.145	14	32	26

Note: ρ=correlation coefficient, unilateral masks used for the amygdala and the hippocampus, bilateral masks used for the medial frontal gyrus and the anterior cingulate gyrus

Table S5. Correlation between true and predicted JDs based on adversities, sex and TIV in the IMAGEN cohort.

**Voxels**	**ρ**	**MAX X (mm)**	**MAX Y (mm)**	**MAX Z (mm)**	**Region**
90174	0.762	-10	-12	-16	Brainstem/hippocampus
172	0.238	-34	18	38	Middle Frontal Gyrus
141	0.227	16	-56	72	Lateral Occipital Cortex
93	0.244	36	20	50	Superior Frontal Gyrus
88	0.376	50	26	38	Middle Frontal Gyrus
70	0.161	-10	8	74	Superior Frontal Gyrus
63	0.183	-36	42	-20	Middle Frontal Gyrus
51	0.299	-30	-70	24	Middle Temporal Gyrus
45	0.161	-22	-54	72	Superior Parietal Lobule
44	0.2	22	-82	50	Lateral Occipital Cortex
41	0.172	38	-60	20	Middle Temporal Gyrus
38	0.15	-26	-80	50	Lateral Occipital Cortex
38	0.167	26	-90	38	Cuneus
31	0.139	-18	26	58	Middle Frontal Gyrus
16	0.255	50	52	2	Middle Frontal Gyrus
15	0.337	18	-36	52	Paracentral Lobule
12	0.134	-46	46	20	Middle Frontal Gyrus

Note: ρ=correlation coefficient

Table S6. Correlation between true and predicted JDs based on adversities, sex and TIV in limbic regions in the IMAGEN subsample.

Region of Interest	hemisphere	Voxels	ρ	MAX X (mm)	MAX Y (mm)	MAX Z (mm)
amygdala	l	633	0.745	-12	-12	-16
	r	728	0.701	14	-8	-12
hippocampus	l	1333	0.751	-10	-12	-18
	r	1366	0.674	12	-12	-16
medial frontal gyrus		755	0.674	-8	30	-12
anterior cingulate gyrus		1961	0.602	-8	34	-10
		3	0.123	-10	18	38
		1	0.1	-6	24	36

Note: ρ=correlation coefficient, unilateral masks used for the amygdala and the hippocampus, bilateral masks used for the medial frontal gyrus and the anterior cingulate gyrus

Table S7. Top 2% of the voxels of the correlation between obstetric risks and the predicted morphometric changes of the JD´s

Voxels	ρ	MAX X (mm)	MAX Y (mm)	MAX Z (mm)	Region
positive association					
3865	0.768	0	54	-18	Medial Frontal Gyrus
2776	0.73	62	4	18	Precentral Gyrus
1180	0.692	-6	20	58	Superior Frontal Gyrus.
1175	0.408	38	-80	-46	Cerebellum
1085	0.547	4	-28	62	Precentral Gyrus
934	0.49	-46	-48	-36	Cerebellum
764	0.363	-46	-4	-4	Insula
591	0.344	2	-96	-10	Occipital Pole
562	0.389	26	-64	2	Lingual Gyrus
414	0.313	-34	-12	56	Precentral Gyrus
335	0.344	-28	20	-42	Temporal Pole
222	0.281	28	40	32	Middle Frontal Gyrus
214	0.54	36	36	16	Frontal Pole
162	0.325	0	-46	-40	Brainstem
139	0.351	2	-68	48	Precuneus
130	0.231	32	-54	42	Superior Parietal Lobule
94	0.262	-42	20	46	Middle Frontal Gyrus
79	0.218	-64	2	18	Precentral Gyrus
69	0.287	46	-16	-36	Inferior Temporal Gyrus
65	0.224	12	-52	48	Precuneus
53	0.243	-28	-34	60	Postcentral Gyrus
50	0.262	-12	0	44	Cingulate Gyrus
37	0.224	-58	-30	-26	Inferior Temporal Gyrus
36	0.161	-66	-50	-4	Middle Temporal Gyrus
31	0.117	56	-40	-14	Middle Temporal Gyrus
29	0.212	0	-50	34	Precuneus
27	0.18	-18	28	38	Superior Frontal Gyrus
27	0.205	-36	62	-2	Middle Frontal Gyrus.
26	0.193	-48	2	56	Precentral Gyrus
25	0.224	46	-42	-40	Cerebellum
24	0.262	36	8	32	Middle Frontal Gyrus

23	0.136	40	-76	0	Lateral Occipital Cortex
23	0.18	2	62	20	Frontal Pole
22	0.212	24	-32	72	Postcentral Gyrus
21	0.136	-44	-58	36	Angular Gyrus
20	0.199	-6	-82	18	Cuneus
19	0.174	-22	36	30	Superior Frontal Gyrus
19	0.18	-28	42	34	Middle Frontal Gyrus
19	0.161	-60	-42	34	Supramarginal Gyrus
19	0.193	18	-38	58	Paracentral Lobule
19	0.123	18	-4	74	Superior Frontal Gyrus
18	0.3	-28	32	-26	Middle Frontal Gyrus
17	0.117	-32	-86	20	Middle Occipital Gyrus
17	0.123	-26	-78	-16	Cerebellum
17	0.11	-52	-52	26	Supramarginal Gyrus
16	0.199	26	-12	52	Precentral Gyrus
15	0.123	6	-46	-2	Cerebellum
15	0.123	16	-80	48	Precuneus
15	0.148	-30	4	62	Middle Frontal Gyrus
14	0.3	-22	-52	50	Precuneus
14	0.205	-20	-20	72	Precentral Gyrus
13	0.237	24	-74	-46	Cerebellum
13	0.117	-34	-72	-34	Cerebellum
12	0.174	40	44	-20	Middle Frontal Gyrus
11	0.11	58	-28	34	Inferior Parietal Lobule
11	0.167	42	-6	62	Precentral Gyrus
10	0.199	26	-86	38	Cuneus
10	0.117	2	32	42	Paracingulate Gyrus
10	0.161	0	-64	-20	Cerebellum
10	0.18	0	40	36	Medial Frontal Gyrus
10	0.136	-38	-60	46	Inferior Parietal Lobule.
negative association					
13354	0.844	28	20	-20	Inferior Frontal Gyrus
1053	0.756	-24	-84	12	Middle Occipital Gyrus.
848	0.781	2	34	0	Anterior Cingulate Gyrus

600	0.68	62	-30	44	Supramarginal Gyrus
375	0.794	-46	40	22	Middle Frontal Gyrus
272	0.598	0	-66	-2	Cerebellum
243	0.642	24	-100	-10	Fusiform Gyrus
232	0.61	14	-46	-58	Cerebellum
227	0.648	6	-86	40	Cuneus
196	0.591	-56	-6	44	Precentral Gyrus
85	0.572	10	0	46	Cingulate Gyrus
76	0.528	20	-54	22	Precuneus
75	0.655	30	62	22	Superior Frontal Gyrus
71	0.68	-20	-80	-8	Lingual Gyrus
70	0.598	-36	40	-16	Middle Frontal Gyrus
62	0.541	-64	-28	44	Supramarginal Gyrus
39	0.604	-56	34	-4	Inferior Frontal Gyrus
35	0.509	12	72	8	Frontal Pole
19	0.509	-32	-88	-24	Cerebellum
19	0.427	-10	22	-8	Anterior Cingulate
17	0.427	38	-80	34	Superior Occipital Gyrus
16	0.452	16	-30	38	Cingulate Gyrus
13	0.433	34	-52	68	Superior Parietal Lobule
12	0.408	56	32	24	Middle Frontal Gyrus

Note: ρ=correlation coefficient

Table S8. Correlation between obstetric risks and the predicted morphometric changes of the JD´s in limbic regions.

**Region of Interest**	**hemisphere**	**Voxels**	**ρ**	**MAX X (mm)**	**MAX Y (mm)**	**MAX Z (mm)**
amygdala	l	137	-0.629	-26	-8	-24
	l	2	-0.357	-32	4	-18
	r	170	-0.73	30	-12	-16
	r	5	-0.465	32	6	-24
	r	3	-0.402	24	6	-26
hippocampus	l	69	0.433	-28	-42	8
	l	910	-0.762	-26	-30	-10
	r	37	0.199	22	-40	-8
	r	1	0.104	30	-34	-18
	r	671	-0.813	30	-14	-18
	r	1	-0.37	12	-40	8
medial frontal gyrus		1317	0.768	0	54	40
anterior cingulate gyrus		205	0.313	4	-18	42
		14	0.25	-12	0	22
		2	0.148	12	38	40
		2	0.199	4	22	-10
		1	0.174	-2	42	18
		1	0.104	-2	18	32
		1	0.104	4	4	40
		1	0.104	8	12	40
		1	0.11	-2	12	42
		1	0.104	-2	0	46
		1	0.129	-6	-8	0
		782	-0.781	2	34	46
		52	-0.570	10	0	34
		5	-0.376	-10	4	8
		1	-0.380	14	44	8

Note: ρ=correlation coefficient, unilateral masks used for the amygdala and the hippocampus, bilateral masks used for the medial frontal gyrus and the anterior cingulate gyrus

Table S9. Top 2% of the voxels of the correlation between prenatal maternal stress and the predicted morphometric changes of the JD´s

Voxels	ρ	MAX X (mm)	MAX Y (mm)	MAX Z (mm)	Region
positive association					
3267	0.594	-22	54	-14	Superior Frontal Gyrus.
1309	0.726	-10	24	50	Superior Frontal Gyrus.
1074	0.508	-66	-50	6	Middle Temporal Gyrus.
980	0.346	68	-42	-12	Middle Temporal Gyrus.
581	0.364	-30	2	46	Middle Frontal Gyrus
579	0.283	-48	-80	-8	Middle Occipital Gyrus.
491	0.513	30	-38	-14	Parahippocampal Gyrus
491	0.462	32	-50	44	Superior Parietal Lobule
479	0.341	-44	6	-22	Superior Temporal Gyrus.
431	0.243	-4	8	6	Caudate
269	0.346	-26	-46	-18	Culmen
250	0.295	-68	-8	18	Postcentral Gyrus
218	0.49	-38	-48	64	Superior Parietal Lobule
207	0.358	54	-18	-32	Fusiform Gyrus
172	0.272	6	-42	38	Cingulate Gyrus
154	0.306	32	-88	-18	Declive
135	0.289	50	-2	52	Precentral Gyrus
98	0.427	-46	-46	8	Middle Temporal Gyrus
91	0.197	12	-62	32	Precuneus
90	0.243	-4	-76	-2	Lingual Gyrus
90	0.295	2	-50	-60	Brain Stem
86	0.352	-18	-72	-58	Cerebellum
81	0.231	44	4	-12	Planum Polare
78	0.208	-16	-24	14	.Thalamus
73	0.272	-4	66	18	Superior Frontal Gyrus
69	0.231	32	18	-14	Insula
68	0.289	40	0	34	Precentral Gyrus
59	0.335	-22	-66	64	Superior Parietal Lobule
56	0.306	66	-2	30	Precentral Gyrus
53	0.185	10	-76	10	Cuneus

52	0.162	54	-76	-8	Middle Occipital Gyrus
50	0.237	-12	-98	22	Middle Occipital Gyrus
36	0.272	40	24	-38	Temporal Pole
36	0.168	18	-32	68	Postcentral Gyrus
34	0.266	52	-10	24	Postcentral Gyrus
33	0.179	-50	-52	-44	Cerebellum
30	0.179	10	-60	-46	Cerebellum
29	0.139	24	8	62	Middle Frontal Gyrus
28	0.214	-16	-74	60	Lateral Occipital Cortex
28	0.145	-22	-40	70	Postcentral Gyrus
24	0.145	8	-10	74	Superior Frontal Gyrus
20	0.139	42	-32	64	Postcentral Gyrus
18	0.128	-42	-70	2	Inferior Temporal Gyrus.
18	0.122	-24	48	22	Superior Frontal Gyrus.
18	0.174	-28	2	-46	Superior Temporal Gyrus
18	0.191	-10	-84	16	Cuneus
18	0.116	38	-78	-8	Middle Occipital Gyrus
17	0.179	8	8	30	Cingulate Gyrus
17	0.197	0	-64	-28	Cerebellum
16	0.139	-12	44	34	Medial Frontal Gyrus
14	0.139	12	-10	70	Superior Frontal Gyrus.
14	0.11	-42	-58	32	Superior Temporal Gyrus
11	0.122	6	-42	68	Paracentral Lobule
10	0.151	-54	28	-12	Inferior Frontal Gyrus
negative association					
11952	0.742	70	-20	-20	Inferior Temporal Gyrus
1437	0.736	-52	-60	38	Supramarginal Gyrus
1333	0.69	-10	-22	40	Cingulate Gyrus
470	0.742	-16	-56	0	Lingual Gyrus
318	0.609	2	-82	34	Cuneus
220	0.517	-58	8	36	Precentral Gyrus
177	0.54	-44	14	14	Inferior Frontal Gyrus
148	0.598	-12	54	36	Superior Frontal Gyrus
146	0.54	64	-28	42	Inferior Parietal Lobule
133	0.558	-12	40	30	Medial Frontal Gyrus

101	0.575	-40	46	28	Superior Frontal Gyrus
100	0.523	38	-16	64	Precentral Gyrus
94	0.465	4	40	-30	Orbital Gyrus
83	0.569	20	-52	56	Precuneus
74	0.529	-8	72	2	Superior Frontal Gyrus
66	0.477	-38	-30	-16	Parahippocampal Gyrus
65	0.46	-34	-62	-60	Cerebellum
59	0.465	-8	-44	20	Corpus Callosum
57	0.437	8	-74	-54	Cerebellum
56	0.437	40	18	56	Middle Frontal Gyrus
49	0.442	8	-78	-10	Lingual Gyrus
44	0.402	-16	20	-8	Accumbens
38	0.534	26	-56	68	Superior Parietal Lobule
35	0.511	60	24	22	Inferior Frontal Gyrus
31	0.465	-6	10	68	Superior Frontal Gyrus
28	0.396	-26	-86	36	Cuneus
27	0.632	-32	56	16	Middle Frontal Gyrus
20	0.494	20	-46	58	Precuneus
19	0.46	8	30	34	Cingulate Gyrus
18	0.425	4	-8	68	Medial Frontal Gyrus
18	0.517	6	-28	26	Posterior Cingulate
17	0.517	-26	-32	60	Postcentral Gyrus
16	0.442	-37	-18	40	Precentral Gyrus
12	0.396	10	8	10	Caudate Body
12	0.35	6	-74	52	Precuneus

Note: ρ=correlation coefficient

Table S10. Correlation between prenatal maternal stress and the predicted morphometric changes of the JD´s in limbic regions.

Region of Interest	hemisphere	Voxels	ρ	MAX X (mm)	MAX Y (mm)	MAX Z (mm)
amygdala	l	55	-0.575	-24	-6	-28
	r	50	-0.448	26	-6	-26
	r	32	-0.529	20	6	-24
hippocampus	l	1	0.105	-28	-40	-6
	l	161	-0.575	-24	-6	-28
	l	12	-0.373	-36	-28	-16
	l	6	-0.373	-16	-36	4
	l	2	-0.344	-34	-32	-4
	l	1	-0.327	-20	-26	-16
	r	151	-0.352	28	-34	-10
	r	128	-0.454	26	-6	-28
	r	2	-0.339	20	-34	2
medial frontal gyrus		181	0.295	-14	46	-4
		2	0.128	12	42	-18
		1	0.122	8	56	-20
		91	-0.465	4	40	-30
		6	-0.448	10	56	-2
anterior cingulate gyrus		331	0.295	-4	28	16
		108	0.358	0	18	40
		16	0.179	8	8	30
		6	0.243	-10	28	18
		5	0.145	-10	42	6
		4	0.128	-12	22	28
		2	0.11	4	40	12
		2	0.11	2	-12	42
		1	0.156	0	42	12
		1	0.128	-10	24	22
		1	0.116	12	8	34
		1	0.243	6	-14	36
		1	0.174	6	-14	46
		1	0.105	0	-4	50
		1	0.11	0	0	50
		344	-0.644	8	-2	44
		30	-0.442	-8	40	24
		7	-0.46	8	30	34
		2	-0.385	4	28	32

Note: ρ=correlation coefficient, unilateral masks used for the amygdala and the hippocampus, bilateral masks used for the medial frontal gyrus and the anterior cingulate gyrus

Table S11. Top 2% of the voxels of the correlation between prenatal maternal smoking and the predicted morphometric changes of the JD´s

Voxels	ρ	MAX X (mm)	MAX Y (mm)	MAX Z (mm)	Region
positive association					
2690	0.586	36	-28	-7	Hippocampus
1762	0.462	-16	-70	-50	Cerebellum
1555	0.5	30	-24	52	Precentral Gyrus
1515	0.5	42	12	-16	Insula
1142	0.327	-12	24	42	Cingulate Gyrus
882	0.36	-40	16	-20	Inferior Frontal Gyrus
840	0.295	46	-52	-4	Inferior Temporal Gyrus
717	0.327	0	-46	40	Precuneus
636	0.22	0	-18	70	Medial Frontal Gyrus
610	0.386	-14	42	2	Anterior Cingulate
567	0.22	18	-62	28	Precuneus
525	0.279	-40	-26	40	Postcentral Gyrus
404	0.43	-24	46	-14	Middle Frontal Gyrus
354	0.306	-30	-88	-18	Declive
287	0.225	-6	-76	34	Cuneus
286	0.241	8	-84	-36	Cerebellum
204	0.22	4	-40	-8	Brain Stem
185	0.236	36	-32	68	Postcentral Gyrus
183	0.295	12	-62	32	Precuneus
180	0.23	-46	-64	2	Middle Temporal Gyrus
178	0.22	-62	-58	4	Middle Temporal Gyrus
159	0.198	-46	2	48	Precentral Gyrus
132	0.203	62	-10	26	Precentral Gyrus
121	0.268	-28	-2	52	Middle Frontal Gyrus
119	0.252	-34	-76	10	Middle Occipital Gyrus.
109	0.209	-42	-64	-38	Cerebellum
106	0.333	-42	-50	20	Middle Frontal Gyrus
94	0.273	-14	34	38	Medial Frontal Gyrus
77	0.198	-12	-44	50	Precuneus
73	0.29	-32	-84	-44	Cerebellum
72	0.284	-58	-50	24	Superior Temporal Gyrus
70	0.198	-44	-10	-36	Inferior Temporal Gyrus
69	0.193	-54	-2	-4	Superior Temporal Gyrus
67	0.236	-22	-40	72	Postcentral Gyrus

63	0.187	50	-24	-24	Inferior Temporal Gyrus
58	0.209	-24	44	30	Superior Frontal Gyrus
57	0.193	-52	-62	14	Middle Temporal Gyrus
55	0.3	-34	22	26	Middle Frontal Gyrus
53	0.182	48	-70	28	Middle Temporal Gyrus
48	0.209	-32	20	60	Middle Frontal Gyrus
47	0.257	-12	-58	8	Precuneus
46	0.214	-46	-14	52	Postcentral Gyrus
41	0.182	-60	-16	26	Postcentral Gyrus
40	0.327	-16	-30	2	Thalamus
37	0.203	-56	-24	-26	Inferior Temporal Gyrus
37	0.182	8	32	-28	Rectal Gyrus
35	0.193	16	66	10	Superior Frontal Gyrus
34	0.193	32	24	-24	Inferior Frontal Gyrus
34	0.182	-40	2	-40	Middle Temporal Gyrus
33	0.203	14	48	-18	Medial Frontal Gyrus
32	0.182	-60	10	-12	Superior Temporal Gyrus
32	0.182	64	-30	22	Superior Temporal Gyrus
31	0.187	-38	56	14	Middle Frontal Gyrus
30	0.22	42	-66	-4	Lateral Occipital Cortex
29	0.187	-28	16	62	Middle Frontal Gyrus
27	0.176	-26	-44	48	Superior Parietal Lobule
27	0.187	-20	-80	24	Cuneus
26	0.225	-22	-10	74	Precentral Gyrus
26	0.187	46	-52	-16	Fusiform Gyrus
26	0.279	4	66	10	Frontal Pole
25	0.187	26	22	-28	Inferior Frontal Gyrus
25	0.176	-48	-38	0	Middle Temporal Gyrus
24	0.176	12	-58	60	Precuneus
24	0.203	44	-62	8	Lateral Occipital Cortex
24	0.252	-6	54	42	Frontal Pole
23	0.187	32	-60	-16	Declive
22	0.187	48	-60	-38	Cerebellum
21	0.214	-28	-20	72	Precentral Gyrus
21	0.193	-34	46	26	Middle Frontal Gyrus

21	0.187	-40	-88	-2	Inferior Occipital Gyrus
18	0.182	-64	-16	26	Postcentral Gyrus
18	0.209	6	44	8	Anterior Cingulate
18	0.187	50	0	44	Precentral Gyrus
18	0.225	6	-42	22	Posterior Cingulate
18	0.214	44	-84	14	Middle Occipital Gyrus
18	0.225	14	38	4	Anterior Cingulate
17	0.23	54	-20	-34	Inferior Temporal Gyrus
17	0.182	-32	2	-48	Superior Temporal Gyrus
17	0.193	-6	-76	28	Cuneus
17	0.176	40	16	4	Insula
17	0.187	42	-74	38	Angular Gyrus
17	0.279	-52	26	-2	Inferior Frontal Gyrus
16	0.182	-54	24	32	Middle Frontal Gyrus
16	0.176	42	-54	16	Superior Temporal Gyrus
16	0.193	-50	-20	54	Postcentral Gyrus
15	0.182	44	-14	-44	Inferior Temporal Gyrus,
15	0.203	-54	-30	2	Superior Temporal Gyrus
15	0.171	-54	10	6	Precentral Gyrus
15	0.193	-4	32	38	Medial Frontal Gyrus
15	0.257	32	-50	48	Superior Parietal Lobule
14	0.193	-26	40	-20	Middle Frontal Gyrus
13	0.23	-48	-48	10	Middle Temporal Gyrus
13	0.182	64	-32	-8	Middle Temporal Gyrus
13	0.203	54	-6	14	Central Opercular Cortex
12	0.171	42	-76	-30	Cerebellum
12	0.187	-10	-32	-20	Brain Stem
12	0.182	36	24	10	Frontal Operculum Cortex
11	0.187	-42	-64	40	Angular Gyrus
11	0.176	-36	-92	-2	Inferior Occipital Gyrus
11	0.187	46	-18	-14	Middle Temporal Gyrus
11	0.241	-52	-16	6	Superior Temporal Gyrus
10	0.176	-60	-22	-28	Inferior Temporal Gyrus

10	0.176	30	48	22	Superior Frontal Gyrus
10	0.22	24	-84	-40	Cerebellum
10	0.176	38	28	40	Middle Frontal Gyrus
10	0.284	-20	-24	58	Postcentral Gyrus
negative association					
5046	0.782	-44	-16	62	Postcentral Gyrus
4284	0.75	-32	-96	4	Middle Occipital Gyrus
1307	0.75	-44	-46	-40	Cerebellum
601	0.75	42	-8	-38	Middle Temporal Gyrus
369	0.728	26	42	40	Middle Frontal Gyrus
361	0.594	-24	-50	-6	Parahippocampal Gyrus
326	0.626	-50	-72	-2	Middle Occipital Gyrus
299	0.701	18	-70	-10	Cerebellum
166	0.626	22	-18	-28	Parahippocampal Gyrus
137	0.551	54	18	-30	Superior Temporal Gyrus
113	0.615	6	-28	40	Cingulate Gyrus
101	0.61	-30	48	34	Middle Frontal Gyrus
90	0.594	-4	-30	50	Precuneus
73	0.637	12	-54	56	Precuneus
71	0.561	-16	-78	-6	Lingual Gyrus
69	0.707	-48	16	48	Middle Frontal Gyrus
65	0.551	-8	10	72	Superior Frontal Gyrus
56	0.535	4	-38	6	Corpus Callosum
52	0.524	-14	-14	-34	Cingulate Gyrus
43	0.669	-16	42	50	Superior Frontal Gyrus
30	0.658	-22	16	66	Superior Frontal Gyrus
26	0.524	-40	-78	-36	Cerebellum
25	0.491	-4	-6	40	Cingulate Gyrus
23	0.47	-60	14	26	Inferior Frontal Gyrus
21	0.47	-16	-36	-52	Cerebellum
17	0.486	-52	30	24	Middle Frontal Gyrus
16	0.545	14	36	30	Medial Frontal Gyrus
11	0.529	-38	62	-8	Middle Frontal Gyrus
11	0.448	-12	-2	44	Cingulate Gyrus
10	0.475	-4	12	34	Cingulate Gyrus
10	0.481	2	-16	68	Precentral Gyrus

Note: ρ=correlation coefficient

Table S12. Correlation between prenatal maternal smoking and the predicted morphometric changes of the JD´s in limbic regions.

Region of Interest	hemisphere	Voxels	ρ	MAX X (mm)	MAX Y (mm)	MAX Z (mm)
amygdala	l	0				
	r	0				
hippocampus	l	183	0.392	-32	-30	-8
	l	2	2	0.214	-16	-34
	l	1	1	0.171	-26	-40
	l	17	-0.486	-24	-44	4
	r	230	0.564	32	-28	-4
	r	28	-0.572	24	-16	-26
	r	12	-0.465	28	-40	6
medial frontal gyrus		19	0.193	8	46	-12
		9	0.182	8	32	-28
		9	0.203	-10	54	2
		8	0.203	4	50	-12
		6	0.193	4	32	-28
		6	0.241	-12	48	0
		614	-0.626	2	28	-22
		1	-0.454	2	56	-8
anterior cingulate gyrus		193	0.386	-14	42	4
		62	0.209	0	0	50
		47	0.252	6	20	40
		18	0.209	6	44	8
		11	0.198	14	40	4
		9	0.268	-4	48	14
		5	0.203	-10	22	24
		4	0.182	-8	-2	50
		3	0.176	10	38	4
		2	0.171	8	42	20
		1	0.193	8	40	-2
		1	0.176	8	42	4
		1	0.176	8	28	18
		25	-0.491	-4	-6	40
		10	-0.475	-4	12	34
		9	-0.502	2	-20	40
		2	-0.411	10	-8	40
		1	-0.411	-10	6	40
		1	-0.443	-4	-20	42
		1	-0.427	-12	0	42

Note: ρ=correlation coefficient, unilateral masks used for the amygdala and the hippocampus, bilateral masks used for the medial frontal gyrus and the anterior cingulate gyrus

Table S13. Top 2% of the voxels of the correlation between maternal sensitivity and the predicted morphometric changes of the JD´s

Voxels	ρ	MAX X (mm)	MAX Y (mm)	MAX Z (mm)	Region
positive association					
6660	0.49	16	38	16	Anterior Cingulate
1384	0.618	-48	26	28	Middle Frontal Gyrus
1252	0.52	-50	-10	26	Precentral Gyrus
1029	0.526	54	-34	-18	Inferior Temporal Gyrus,
953	0.556	-36	-12	60	Precentral Gyrus
802	0.49	-26	-76	48	Precuneus
607	0.356	30	50	38	Superior Frontal Gyrus
589	0.336	2	-80	-6	Lingual Gyrus
496	0.474	42	-82	-32	Cerebellum
486	0.361	-50	16	-20	Superior Temporal Gyrus
444	0.51	-42	-82	-30	Cerebellum
438	0.387	64	-44	30	Cingulate Gyrus
414	0.315	38	4	18	Insula
370	0.341	30	-74	46	Precuneus
293	0.32	-8	-62	-54	Cerebellum
280	0.336	10	-98	14	Cuneus
126	0.408	14	-34	80	Postcentral Gyrus
95	0.274	14	-50	28	Posterior Cingulate
86	0.556	30	-40	40	Superior Parietal Lobule
86	0.372	44	-48	10	Middle Temporal Gyrus.
62	0.295	-54	-32	-20	Inferior Temporal Gyrus
60	0.382	46	-32	36	Inferior Parietal Lobule
51	0.295	12	-82	24	Cuneus
48	0.244	-38	-96	8	Middle Occipital Gyrus
45	0.274	34	-16	48	Precentral Gyrus.
42	0.187	-68	-30	-10	Middle Temporal Gyrus
42	0.187	-50	-68	-8	Middle Occipital Gyrus
41	0.244	42	-50	50	Inferior Parietal Lobule
34	0.438	4	12	70	Superior Frontal Gyrus

33	0.228	-8	16	44	Medial Frontal Gyrus
31	0.29	64	-6	32	Precentral Gyrus
30	0.203	14	60	38	Frontal Pole
27	0.177	-44	-86	26	Middle Temporal Gyrus
25	0.192	12	-80	-50	Cerebellum
23	0.315	46	-4	28	Precentral Gyrus
18	0.187	10	-64	-36	Cerebellum
14	0.341	34	14	26	Middle Frontal Gyrus
10	0.177	-42	32	-8	Inferior Frontal Gyrus
10	0.156	18	-58	-28	Cerebellum
negative association					
8516	0.648	-36	-26	-24	Parahippocampal Gyrus
1941	0.689	-68	-22	6	Superior Temporal Gyrus
1175	0.582	10	-14	56	Medial Frontal Gyrus
1050	0.582	34	-64	-56	Cerebellum
721	0.535	52	-72	20	Middle Temporal Gyrus
701	0.494	0	-66	56	Precuneus
501	0.525	-42	20	-24	Temporal Pole
307	0.469	42	12	52	Middle Frontal Gyrus
305	0.551	-6	8	36	Cingulate Gyrus
266	0.479	-60	-42	30	Inferior Parietal Lobule
210	0.407	30	-100	6	Middle Occipital Gyrus
193	0.443	4	54	-2	Cerebellum
183	0.484	-2	40	36	Medial Frontal Gyrus
146	0.397	-34	-72	20	Middle Occipital Gyrus
132	0.351	-8	10	60	Medial Frontal Gyrus
109	0.356	6	-96	-10	Lingual Gyrus
92	0.474	38	62	8	Superior Frontal Gyrus
88	0.515	-32	-48	70	Superior Parietal Lobule
75	0.407	-16	20	-20	Frontal Orbital Cortex
52	0.371	-26	64	-4	Superior Frontal Gyrus

35	0.31	-8	34	50	Superior Frontal Gyrus
32	0.366	28	-8	-18	Amygdala
28	0.418	34	6	44	Middle Frontal Gyrus
27	0.31	-44	-84	12	Middle Occipital Gyrus
26	0.402	-26	-32	60	Postcentral Gyrus
25	0.361	62	-52	38	Supramarginal Gyrus
24	0.259	-6	-60	-42	Cerebellum
23	0.325	8	30	58	Superior Frontal Gyrus
21	0.295	-36	34	-16	Inferior Frontal Gyrus
20	0.284	26	-62	66	lateral Occipital Cortex
20	0.387	-8	-18	78	Precentral Gyrus
19	0.269	44	-16	60	Precentral Gyrus
18	0.284	-22	24	62	Superior Frontal Gyrus
15	0.31	10	66	8	Medial Frontal Gyrus
13	0.371	-40	12	60	Middle Frontal Gyrus
12	0.346	-24	-48	46	Parietal Lobe

Note: ρ=correlation coefficient

Table S14. Correlation between maternal sensitivity and the predicted morphometric changes of the JD´s in limbic regions.

Region of Interest	hemisphere	Voxels	ρ	MAX X (mm)	MAX Y (mm)	MAX Z (mm)
amygdala	l	79	0.305	-22	0	-30
	l	14	-0.305	-30	-12	-18
	l	3	-0.223	-12	-2	-18
	l	2	-0.238	-16	0	-16
	r	153	0.341	28	-2	-28
	r	22	-0.366	28	-8	-18
hippocampus	l	122	0.29	-34	-26	-8
	l	40	0.264	-22	-4	-28
	l	70	-0.412	-16	-34	-8
	l	38	-0.305	-34	-22	-22
	r	285	0.341	28	-2	-28
	r	1	96	0.197	28	-38
	r	46	-0.402	20	-32	-4
	r	25	-0.366	28	-8	-18
	r	5	-0.259	30	-32	-14
	r	3	-0.223	32	-28	-6
	r	3	-0.254	8	-40	6
medial frontal gyrus		267	0.315	-8	52	-14
		97	0.213	12	36	-18
		1	0.151	-6	58	-2
		65	-0.443	4	54	-2
		3	-0.233	0	44	-28
		2	-0.248	2	36	-30
		2	-0.238	-2	28	-28
		1	-0.218	0	28	-24
anterior cingulate gyrus		1339	0.469	14	36	20
		9	0.187	-4	14	44
		5	0.197	-10	24	22
		2	0.192	4	14	40
		298	-0.551	-6	8	36
		96	-0.412	-2	36	30
		33	-0.407	8	-10	50
		6	-0.3	2	-22	30
		2	-0.238	-4	-20	44
		2	-0.254	2	-18	46

Note: ρ=correlation coefficient, unilateral masks used for the amygdala and the hippocampus, bilateral masks used for the medial frontal gyrus and the anterior cingulate gyrus

Table S15. Top 2% of the voxels of the correlation between psychosocial family risks and the predicted morphometric changes of the JD´s

Voxels	ρ	MAX X (mm)	MAX Y (mm)	MAX Z (mm)	Region
positive association					
7746	0.597	46	50	6	Middle Frontal Gyrus
7528	0.575	-18	-68	34	Precuneus
1319	0.432	-68	-46	-8	Middle Temporal Gyrus
533	0.514	-32	40	44	Middle Frontal Gyrus
363	0.602	-34	0	58	Middle Frontal Gyrus
276	0.333	14	-84	18	Cuneus
261	0.415	64	-46	28	Supramarginal Gyrus
143	0.206	22	2	-42	Fusiform Gyrus
118	0.465	26	-22	56	Postcentral Gyrus
103	0.536	-34	-50	66	Superior Parietal Lobule
77	0.267	32	34	50	Superior Frontal Gyrus
43	0.206	2	-28	-12	Brainstem
42	0.233	16	-40	-44	Cerebellum
39	0.349	-44	-48	20	Superior Temporal Gyrus
34	0.377	-28	28	30	Middle Frontal Gyrus
33	0.189	28	-44	40	Superior Parietal Lobule
28	0.184	-64	-32	0	Middle Temporal Gyrus
27	0.173	26	-60	-36	Cerebellum
24	0.211	32	-96	-14	Fusiform Gyrus
24	0.228	-38	-34	68	Postcentral Gyrus
20	0.228	34	-50	66	Superior Parietal Lobule
19	0.256	-22	-22	56	Postcentral Gyrus
16	0.311	46	-50	-4	Inferior Temporal Gyrus
16	0.305	62	-34	-20	Inferior Temporal Gyrus
15	0.338	48	-40	28	Inferior Parietal Lobule
13	0.195	16	-38	-32	Pons
12	0.162	2	66	14	Frontal Pole

12	0.189	2	40	52	Superior Frontal Gyrus
10	0.167	2	-86	38	Precuneus
10	0.189	28	-68	-24	Cerebellum
negative association					
8566	0.801	-2	-48	-42	Brain Stem
2016	0.741	54	-30	12	Superior Temporal Gyrus
1167	0.603	-40	-20	58	Postcentral Gyrus
900	0.774	-4	62	0	Medial Frontal Gyrus
744	0.724	-50	-68	26	Middle Temporal Gyrus
632	0.641	-12	-46	48	Precuneus
504	0.63	-20	-60	4	Lingual Gyrus
489	0.746	-36	12	-38	Superior Temporal Gyrus
359	0.526	50	-34	-27	Inferior Temporal Gyrus
338	0.763	-4	10	66	Superior Frontal Gyrus
161	0.57	20	50	44	Superior Frontal Gyrus
141	0.482	-30	-84	-36	Cerebellum
137	0.531	-10	-64	68	Lateral Occipital Cortex
116	0.63	42	-14	-38	Inferior Temporal Gyrus
108	0.614	10	-52	56	Precuneus
100	0.471	44	30	-20	Inferior Frontal Gyrus
98	0.548	-42	20	50	Middle Frontal Gyrus
96	0.438	56	16	-26	Superior Temporal Gyrus
76	0.553	-44	8	2	Insula
52	0.526	6	-52	12	Posterior Cingulate
41	0.46	30	16	-36	Superior Temporal Gyrus
35	0.438	40	42	30	Superior Frontal Gyrus
33	0.394	48	20	36	Precentral Gyrus
30	0.41	40	-92	6	Middle Occipital Gyrus
29	0.421	2	-18	66	Precentral Gyrus
28	0.427	-18	20	-24	Inferior Frontal Gyrus
24	0.399	8	-70	-28	Cerebellum
20	0.449	50	-46	46	Inferior Parietal Lobule

17	0.46	-24	40	22	Frontal Pole
16	0.427	30	-72	-46	Cerebellum
16	0.394	24	-50	-18	Cerebellum
16	0.311	-58	-64	-8	Inferior Temporal Gyrus
13	0.542	-24	-48	46	Superior Parietal Lobule
12	0.377	-26	-34	60	Postcentral Gyrus
11	0.311	62	-42	50	Inferior Parietal Lobule
10	0.432	-32	64	0	Middle Frontal Gyrus
10	0.322	-16	52	30	Superior Frontal Gyrus
10	0.399	-28	-10	72	Precentral Gyrus

Note: ρ=correlation coefficient

Table S16. Correlation between psychosocial family risks and the predicted morphometric changes of the JD´s in limbic regions.

Region of Interest	hemisphere	Voxels	ρ	MAX X (mm)	MAX Y (mm)	MAX Z (mm)
amygdala	l	261	0.305	-32	-2	-26
	r	90	0.25	18	-10	-10
	r	3	0.14	34	2	-22
hippocampus	l	404	0.399	-32	-28	-6
	l	4	0.245	-32	-4	-26
	l	1	0.156	-26	-2	-28
	l	36	-0.531	-26	-44	2
	l	1	-0.295	-22	-14	-28
	r	236	0.404	26	-32	-12
	r	12	0.344	14	-40	8
	r	4	0.184	18	-14	-12
	r	1	96	0.197	28	-38
	r	5	-0.361	24	-16	-26
	r	1	-0.289	26	-42	4
medial frontal gyrus		30	0.305	12	42	-18
		3	0.167	-12	48	0
		1	0.145	-12	40	-4
		318	-0.63	-4	58	-2
		1	-0.306	8	46	-10
anterior cingulate gyrus		1523	0.542	-12	42	2
		3	0.217	16	44	4
		1	0.145	14	40	4
		1	0.173	2	32	26
		143	-0.471	-4	-20	42
		7	-0.333	0	-16	42
		1	-0.284	2	-4	40

Note: ρ=correlation coefficient, unilateral masks used for the amygdala and the hippocampus, bilateral masks used for the medial frontal gyrus and the anterior cingulate gyrus

Table S17. Top 2% of the voxels of the correlation between trauma and the predicted morphometric changes of the JD´s

Voxels	ρ	MAX X (mm)	MAX Y (mm)	MAX Z (mm)	Region
positive association					
4462	0.575	-50	-20	-16	Middle Temporal Gyrus
2699	0.484	-14	46	2	Anterior Cingulate
1792	0.489	46	26	32	Precentral Gyrus
1136	0.479	26	-28	-16	Parahippocampal Gyrus
318	0.371	-26	16	42	Middle Frontal Gyrus
252	0.258	62	-6	38	Precentral Gyrus
169	0.452	28	-48	50	Precuneus
168	0.339	-44	52	18	Middle Frontal Gyrus
132	0.312	60	-42	-18	Inferior Temporal Gyrus
120	0.29	-60	-64	0	Middle Temporal Gyrus
94	0.242	-30	-66	52	Superior Parietal Lobule
91	0.312	28	-16	56	Precentral Gyrus
88	0.172	-40	-56	54	Superior Parietal Lobule
85	0.29	6	-44	50	Precuneus
73	0.231	-36	36	34	Middle Frontal Gyrus
72	0.236	26	36	50	Superior Frontal Gyrus
57	0.129	36	-34	58	Inferior Parietal Lobule
57	0.161	-16	-84	-18	Declive
56	0.188	44	10	-4	Insula
53	0.156	0	-2	60	Medial Frontal Gyrus
53	0.134	-32	-40	66	Postcentral Gyrus
52	0.226	-32	-24	46	Postcentral Gyrus
52	0.193	58	-60	32	Superior Temporal Gyrus
43	0.156	22	58	0	Superior Frontal Gyrus.
40	0.123	52	-44	14	Superior Temporal Gyrus
37	0.134	-32	-72	-36	Cerebellum
36	0.199	12	-64	28	Precuneus
32	0.161	-6	-62	-2	Cerebellum

31	0.134	-20	-66	50	Precuneus
31	0.177	20	14	18	Caudate
28	0.226	-40	-30	34	Inferior Parietal Lobule
27	0.156	32	8	-48	Temporal Pole
26	0.129	-4	-42	64	Postcentral Gyrus
26	0.193	-52	-34	32	Inferior Parietal Lobule
26	0.145	-10	-64	46	Precuneus
25	0.166	-4	-12	64	Medial Frontal Gyrus
25	0.177	32	-54	62	Superior Parietal Lobule
24	0.231	-18	12	20	Caudate
23	0.118	46	-60	42	Inferior Parietal Lobule
23	0.113	60	-34	24	Inferior Parietal Lobule
22	0.129	-22	-88	-20	Cerebellum
22	0.231	18	-18	70	Precentral Gyrus
22	0.183	42	-8	36	Precentral Gyrus
21	0.172	32	14	-20	Inferior Frontal Gyrus
21	0.22	28	-30	72	Postcentral Gyrus
20	0.14	44	-82	28	Middle Temporal Gyrus
20	0.145	52	-36	-6	Middle Temporal Gyrus
19	0.123	36	2	64	Middle Frontal Gyrus
19	0.129	-10	-44	66	Postcentral Gyrus
18	0.172	62	-22	26	Inferior Parietal Lobule
18	0.118	-30	-68	-18	Cerebellum
17	0.156	0	-46	44	Precuneus
17	0.193	50	-50	-4	Middle Temporal Gyrus
16	0.15	46	16	40	Middle Frontal Gyrus
15	0.156	-12	-96	-14	Occipital Pole
15	0.231	20	26	40	Superior Frontal Gyrus
15	0.118	-14	-40	70	Postcentral Gyrus
15	0.134	-56	4	32	Precentral Gyrus
15	0.231	-58	-20	48	Postcentral Gyrus
15	0.15	-4	-86	-22	Cerebellum
15	0.107	-56	6	-6	Superior Temporal Gyrus
15	0.123	-10	-10	70	Superior Frontal Gyrus

14	0.134	-14	50	42	Superior Frontal Gyrus
13	0.113	56	-48	6	Middle Temporal Gyrus
13	0.183	44	-8	-50	Inferior Temporal Gyrus
12	0.107	42	-74	-8	Middle Occipital Gyrus
12	0.215	50	-44	0	Middle Temporal Gyrus
12	0.183	2	40	52	Superior Frontal Gyrus
12	0.118	38	24	2	Insula
11	0.134	-68	-50	-8	Middle Temporal Gyrus
11	0.145	-62	-2	26	Precentral Gyrus
11	0.113	22	-72	52	Precuneus
11	0.107	26	4	62	Middle Frontal Gyrus
11	0.193	42	2	56	Middle Frontal Gyrus
10	0.15	-48	-46	52	Inferior Parietal Lobule
10	0.123	48	-26	58	Postcentral Gyrus
10	0.145	-8	-88	38	Cuneus
10	0.118	-20	8	-46	Temporal Pole
10	0.14	44	-30	62	Postcentral Gyrus
10	0.102	34	-74	-12	Fusiform Gyrus
10	0.177	-14	-84	40	Cuneus
10	0.113	42	-62	-10	Lateral Occipital Cortex
negative association					
8376	0.797	8	-50	-46	Brainstem
1360	0.711	60	-42	10	Superior Temporal Gyrus
1173	0.759	-64	-14	8	Superior Temporal Gyrus
1132	0.668	-18	-102	6	Cuneus
521	0.657	32	16	-38	Superior Temporal Gyrus
507	0.716	-12	62	24	Superior Frontal Gyrus
409	0.555	12	52	-26	Medial Frontal Gyrus
332	0.732	38	44	30	Superior Frontal Gyrus
291	0.625	-16	40	50	Superior Frontal Gyrus
290	0.49	-64	-56	14	Superior Temporal Gyrus

237	0.662	-2	-30	54	Precentral Gyrus
192	0.678	14	-52	56	Precuneus
158	0.565	-46	-24	60	Postcentral Gyrus
149	0.522	50	-42	52	Inferior Parietal Lobule
144	0.565	48	-18	62	Postcentral Gyrus
129	0.533	28	-84	42	Cuneus
126	0.506	-40	10	-4	Insula
113	0.452	40	-74	4	Lateral Occipital Cortex
97	0.549	-46	-60	54	Lateral Occipital Cortex
63	0.485	8	-66	-6	Lingual Gyrus
52	0.495	42	-10	-34	Inferior Temporal Gyrus
51	0.463	-30	-74	46	Precuneus
47	0.49	0	-68	52	Precuneus
45	0.555	4	66	24	Frontal Pole
42	0.442	-56	12	-24	Superior Temporal Gyrus
35	0.463	62	-40	50	nferior Parietal Lobule
30	0.458	-50	-72	-2	Middle Occipital Gyrus
29	0.452	-12	20	-28	Frontal Orbital Cortex
29	0.517	0	-14	66	Medial Frontal Gyrus
24	0.517	-12	-48	48	Precuneus
19	0.49	18	-30	68	Precentral Gyrus
17	0.452	-34	40	-8	Frontal Pole
14	0.538	-16	-66	66	Lateral Occipital Cortex
11	0.522	-30	-84	-38	Cerebellum
10	0.425	-46	18	48	Middle Frontal Gyrus

Note: ρ=correlation coefficient

Table S18. Correlation between trauma and the predicted morphometric changes of the JD´s in limbic regions.

Region of Interest	hemisphere	Voxels	ρ	MAX X (mm)	MAX Y (mm)	MAX Z (mm)
amygdala	l	55	0.161	-30	2	-20
	r	10	0.166	24	6	-24
	r	2	0.129	28	6	-24
	r	1	0.102	26	2	-12
hippocampus	l	428	0.446	-26	-38	-8
	l	1	0.102	-38	-24	-12
	l	10	-0.452	-24	-44	4
	l	2	-0.377	-24	-6	-32
	r	475	0.473	28	-36	-8
medial frontal gyrus		21	0.29	-12	48	0
		3	0.145	-10	56	-6
		2	0.113	12	50	0
		1	0.102	-14	30	-16
		292	-0.544	4	42	-30
		4	-0.377	6	54	-8
		2	-0.361	-2	50	-10
		2	-0.399	-4	56	-10
anterior cingulate gyrus		1868	0.425	-12	48	2
		5	0.15	6	-10	50
		3	0.134	2	-4	46
		2	0.107	-4	-10	50
		1	0.113	-2	38	10
		1	0.113	-4	30	16
		1	0.102	0	30	18
		1	0.102	-4	0	44
		1	0.118	6	-8	46

Note: ρ=correlation coefficient, unilateral masks used for the amygdala and the hippocampus, bilateral masks used for the medial frontal gyrus and the anterior cingulate gyrus

Table S19. Top 2% of the voxels of the correlation between stressful life events and the predicted morphometric changes of the JD´s

Voxels	ρ	MAX X (mm)	MAX Y (mm)	MAX Z (mm)	Region
positive association					
12469	0.647	-16	-58	20	Precuneus
2441	0.653	2	20	40	Cingulate Gyrus
656	0.452	-62	-48	18	Superior Temporal Gyrus
371	0.452	40	14	-46	Temporal Pole
364	0.47	14	-34	58	Postcentral Gyrus
213	0.458	-42	28	34	Middle Frontal Gyrus
212	0.33	-26	4	-50	Superior Temporal Gyrus
186	0.482	58	-20	-34	Inferior Temporal Gyrus
121	0.361	46	-52	0	Middle Temporal Gyrus
119	0.385	18	-60	34	Precuneus
104	0.391	32	-50	46	Superior Parietal Lobule
93	0.275	68	-42	18	Superior Temporal Gyrus
76	0.409	-44	-48	12	Superior Temporal Gyrus
60	0.294	60	-36	-22	Fusiform Gyrus
57	0.367	-22	-14	54	Precentral Gyrus
56	0.294	0	-28	32	Cingulate Gyrus
55	0.342	-6	30	-28	Rectal Gyrus
51	0.275	12	-84	22	Cuneus
42	0.251	18	-66	-46	Cerebellum
27	0.257	-30	24	40	Middle Frontal Gyrus
24	0.269	46	-14	32	Postcentral Gyrus
21	0.318	-40	-24	48	Postcentral Gyrus
21	0.354	-16	-70	-48	Cerebellum
19	0.336	-40	56	18	Superior Frontal Gyrus
19	0.281	-18	-84	-4	Lingual Gyrus
16	0.251	-14	-44	-42	Cerebellum
13	0.324	-20	-28	56	Precentral Gyrus
10	0.227	-54	-52	-46	Cerebellum
10	0.227	-34	-50	66	Superior Parietal Lobule
negative association					
9710	0.9	-44	-60	-42	Cerebellum
1406	0.741	50	-72	40	Precuneus
1044	0.62	4	54	-8	Anterior Cingulate Cortex
931	0.784	54	-20	16	Insula
611	0.76	38	-44	-50	Cerebellum
519	0.857	20	52	40	Superior Frontal Gyrus

482	0.711	-4	-34	50	Precuneus
463	0.76	10	-52	56	Precuneus
379	0.802	38	-16	66	Precentral Gyrus
291	0.626	48	-32	-28	Inferior Temporal Gyrus
224	0.54	-66	-18	-14	Middle Temporal Gyrus
118	0.522	-32	14	-38	Superior Temporal Gyrus
113	0.589	42	-14	-38	Inferior Temporal Gyrus
97	0.516	-16	42	50	Superior Frontal Gyrus
91	0.626	2	-6	66	Supplementary Motor Cortex
86	0.38	22	-14	-34	Parahippocampal Gyrus
85	0.41	-16	-40	-54	Cerebellum
83	0.51	-12	-22	17	Thalamus
79	0.565	-34	46	32	Middle Frontal Gyrus
57	0.614	-44	10	6	Frontal Operculum Cortex
57	0.516	36	24	-4	Insula
55	0.516	28	16	-36	Superior Temporal Gyrus
51	0.467	-4	10	62	Medial Frontal Gyrus
48	0.455	-10	0	42	Cingulate Gyrus
47	0.42	39	14	14	Frontal Operculum Cortex
35	0.474	-44	40	-14	Middle Frontal Gyrus
26	0.443	26	-72	-42	Cerebellum
24	0.467	62	4	32	Precentral Gyrus
21	0.425	48	30	-16	Inferior Frontal Gyrus
19	0.346	62	-4	10	Central Opercular Cortex
19	0.394	-28	-36	-40	Cerebellum
17	0.492	-22	40	22	Frontal Pole
14	0.376	-10	26	64	Superior Frontal Gyrus
14	0.431	-22	52	34	Superior Frontal Gyrus
12	0.382	4	-60	20	Precuneus
10	0.358	-54	6	8	Precentral Gyrus

Note: ρ=correlation coefficient

Table S20. Correlation between stressful life events and the predicted morphometric changes of the JD´s in limbic regions.

Region of Interest	hemisphere	Voxels	ρ	MAX X (mm)	MAX Y (mm)	MAX Z (mm)
amygdala	l	537	0.555	-32	4	-18
	r	241	0.348	32	6	-22
hippocampus	l	789	0.519	-36	-16	-16
	l	11	0.452	-24	-44	4
	l	5	0.377	-24	-6	-32
	l	45	-0.687	-24	-44	4
	r	373	0.531	34	-26	-10
	r	6	0.263	16	-38	6
	r	2	-0.34	26	-40	6
medial frontal gyrus		24	0.342	-6	30	-28
		21	0.239	12	38	-18
		3	0.19	-12	48	0
		1	0.184	-12	40	-4
		312	-0.62	4	54	-8
		3	-0.34	8	40	-26
		2	-0.352	2	32	-22
anterior cingulate gyrus		1521	0.653	2	20	40
		48	-0.455	-10	0	42
		3	-0.407	-4	-20	44

Note: ρ=correlation coefficient, unilateral masks used for the amygdala and the hippocampus, bilateral masks used for the medial frontal gyrus and the anterior cingulate gyrus

Table S21. Dice coefficients

Dice coefficients	*Obstetric risks*	*Prenatal maternal stress*	*Prenatal smoke*	*Maternal sensitivity*	*Psycho-social family risks*	*Trauma*	*Stressfull life events*
Obstetric risks	1	0.26	0.03	0.15	0.08	0.07	0.08
Prenatal maternal stress	0.26	1	0.1	0.19	0.24	0.17	0.11
Prenatal smoke	0.03	0.1	1	0.02	0.09	0.19	0.17
Maternal sensitivity	0.15	0.19	0.02	1	0.24	0.15	0.19
Psychosocial family risks	0.08	0.24	0.09	0.24	1	0.32	0.54
Trauma	0.07	0.17	0.19	0.15	0.32	1	0.34
Stressfull life events	0.08	0.11	0.17	0.19	0.54	0.34	1
**Mean Coefficient**	0.24	0.30	0.23	0.28	0.36	0.32	0.35

Table S22. Rotated component loadings.

	PC1	PC2	PC3
Maternal smoking during pregnancy	**0.45478384**	0.01501976	-0.32874476
Prenatal maternal stress	0.274652	**0.62328514**	0.17805955
Early mother-child interaction	0.06032054	-0.01171885	**0.91377406**
Obstetric adversity	-0.09027238	**0.74860608**	-0.10826119
Psychosocial family adversity	**0.52159315**	0.02730697	0.04837519
Childhood trauma	**0.39768412**	-0.2098639	0.09292793
Life events	**0.52510779**	0.07715655	0.05042219

Table S23. Correlation between true and predicted modulated grey matter volume based on adversities, sex and TIV at T1.

Voxels	ρ	MAX X (mm)	MAX Y (mm)	MAX Z (mm)	Region
69912	0.628	20	-32	-18	Parahippocampal Gyrus
1318	0.251	2	-78	28	Cuneus
763	0.256	-50	-52	6	Middle Temporal Gyrus
340	0.247	-34	-92	10	Middle Occipital Gyrus
294	0.238	-38	-50	44	Inferior Parietal Lobule
256	0.184	-60	-42	48	Supramarginal Gyrus
158	0.162	16	2	-48	Temporal Fusiform Cortex
43	0.171	66	-30	30	Supramarginal Gyrus
34	0.126	-64	-4	-30	Middle Temporal Gyrus
28	0.139	-6	-98	-20	Occipital Pole
25	0.171	-6	-76	48	Precuneus
25	0.144	46	-44	12	Superior Temporal Gyrus
15	0.135	-30	-60	48	Lateral Occipital Cortex
14	0.112	-60	-20	-8	Middle Temporal Gyrus.
10	0.126	32	-34	72	Postcentral Gyrus

Note: ρ=correlation coefficient

Table S24. Correlation between true and predicted modulated grey matter volume based on adversities, sex and TIV at T1 in limbic regions.

Region of Interest	hemisphere	Voxels	ρ	MAX X (mm)	MAX Y (mm)	MAX Z (mm)
amygdala	l	552	0.516	-26	-16	-14
	r	594	0.516	28	-16	-12
hippocampus	l	1267	0.566	-18	-34	-4
	r	1195	0.624	34	-24	-12
medial frontal gyrus		1811	0.476	12	40	-18
		1	0.108	4	58	-22
anterior cingulate gyrus		3953	0.404	-12	48	8
		6	0.126	10	-16	38

Note: ρ=correlation coefficient, unilateral masks used for the amygdala and the hippocampus, bilateral masks used for the medial frontal gyrus and the anterior cingulate gyrus

Table S25. Sample description and demographics.

	N (subjects)	N (sites)	Sex (%F/%M)	Mean age (SD)
**All**	19.759	9	53.2/46.8	57.33 (16.96)
**Training set**	9.859	9	53.7/46.3	57.6 (16.80)
**Test set**	9.900	9	52.6/47.4	57.06 (17.11)

Table S26. Demographics across sites.

Site	N	Sex (F%/M%)	Mean age (SD)	Age range
Cam-CAN	656	50.6/49.4	54.93 (18.60)	18-89
HCP	1112	54.5/45.5	28.80 (3.70)	22-37
IMAGEN	115	56.5/43.5	22	22
MARS 1	169	58.6/41.4	25	25
MARS 2	114	60.5/39.5	33	33
OASIS 3	2144	56.8/43.2	70.60(9.5)	43-97
PNC	1296	51.9/48.1	14.37(3.45)	8-21
UKB-11025.0	12008	52.1/47.9	62.34(7.47)	44-88
UKB-11027.0	2145	55/45	63.19 (7.44)	47-88

Table S27. Explained variance of normative model based on age, sex and site.

Voxels	EV	MAX X (mm)	MAX Y (mm)	MAX Z (mm)	Region
77399	0.584	-28	40	-22	Middle Frontal Gyrus
354	0.262	28	-36	44	Postcentral Gyrus
232	0.202	8	44	56	Frontal Pole
164	0.173	42	12	60	Middle Frontal Gyrus
115	0.154	18	20	68	Superior Frontal Gyrus
48	0.142	-60	-28	50	Inferior Parietal Lobule
45	0.139	12	-10	78	Superior Frontal Gyrus
44	0.138	-50	-86	-2	Inferior Occipital Gyrus
31	0.153	56	20	32	Middle Frontal Gyrus
29	0.113	-10	-10	78	Superior Frontal Gyrus
25	0.139	-50	-54	56	Supramarginal Gyrus
24	0.119	0	30	4	Corpus Callosum
16	0.114	16	-16	40	Cingulate Gyrus
13	0.165	10	-34	80	Postcentral Gyrus

Note: EV=explained variance

Table S28. Explained variance of normative model based on age, sex and site in limbic regions.

Region of Interest	hemisphere	Voxels	EV	MAX X (mm)	MAX Y (mm)	MAX Z (mm)
amygdala	l	641	0.297	-12	-12	-16
	r	728	0.309	12	-10	-18
hippocampus	l	1374	0.374	-20	-42	6
	r	1381	0.364	20	-42	6
medial frontal gyrus		1920	0.477	-4	28	-28
anterior cingulate gyrus		4876	0.301	-6	-14	28

Note: EV=explained variance

Table S29. Top 2% of the voxels of the correlation between age and the predicted morphometric changes of the JD´s

Voxels	ρ	MAX X (mm)	MAX Y (mm)	MAX Z (mm)	Region
positive association					
10715	0.927	2	-84	-26	Cerebellum
1479	0.905	-58	-12	42	Precentral Gyrus
1152	0.824	54	-26	54	Postcentral Gyrus
896	0.883	-46	12	-8	Superior Temporal Gyrus
833	0.839	34	-84	36	Cuneus
801	0.817	-24	-90	32	Cuneus
790	0.817	48	16	-10	Inferior Frontal Gyrus
538	0.765	44	-40	-46	Cerebellum
372	0.707	20	16	-40	Temporal Pole
364	0.817	56	18	30	Middle Frontal Gyrus
107	0.567	-36	-58	18	Middle Temporal Gyrus
89	0.611	-64	-22	12	Superior Temporal Gyrus
54	0.64	68	-20	18	Postcentral Gyrus
36	0.574	-2	-38	54	Paracentral Lobule
16	0.442	28	-92	-34	Cerebellum
13	0.435	36	-24	24	Parietal Operculum Cortex
negative association					
4051	0.933	48	42	4	Inferior Frontal Gyrus
1982	0.911	-56	-54	20	Superior Temporal Gyrus
1975	0.896	0	-10	36	Cingulate Gyrus
1230	0.933	-26	-72	-18	Cerebellum
1193	0.947	28	-74	-18	Cerebellum
825	0.889	54	-50	36	Supramarginal Gyrus
351	0.874	20	-68	-56	Cerebellum
312	0.852	50	-30	-4	Middle Temporal Gyrus
263	0.793	-12	22	56	Superior Frontal Gyrus
210	0.786	-54	-32	38	nferior Parietal Lobule.
200	0.815	-18	-68	-56	Cerebellum
141	0.918	-12	-22	6	Thalamus
126	0.918	16	-22	4	Thalamus
121	0.859	-24	-42	-14	Parahippocampal Gyrus
112	0.771	48	-18	-32	Fusiform Gyrus
103	0.844	18	12	-6	Putamen

87	0.822	-18	10	-6	Putamen
73	0.749	-28	-8	52	Precentral Gyrus
66	0.727	-52	-14	-38	Inferior Temporal Gyrus

Note: ρ=correlation coefficient

Table S30. Top 2% of the voxels of the correlation between age and the predicted morphometric changes of the JD´s in limbic regions.

Region of Interest	hemisphere	Voxels	ρ	MAX X (mm)	MAX Y (mm)	MAX Z (mm)
amygdala	l	3	0.442	-10	-4	-20
	r	3	0.405	10	-4	-20
hippocampus	l	205	0.699	-16	-32	-10
	l	146	0.824	-24	-44	4
	r	401	0.817	24	-40	8
medial frontal gyrus		337	-0.837	2	50	-2
		209	-0.859	-2	38	-22
anterior cingulate gyrus		223	-0.844	4	48	2
		206	-0.896	0	-10	36
		7	-0.734	2	32	6
		649	0.868	0	-6	26

Note: ρ=correlation coefficient, unilateral masks used for the amygdala and the hippocampus, bilateral masks used for the medial frontal gyrus and the anterior cingulate gyrus
