# Supplementary material for: A stable and replicable neural signature of lifespan adversity in the adult brain
Source: Nat Neurosci. 2023 Aug 21;26(9):1603–12. doi: 10.1038/s41593-023-01410-8 (PMC10471497; doi:10.1038/s41593-023-01410-8)
Supplement: Supplementary file 2 — Reporting Summary [file 41593_2023_1410_MOESM2_ESM.pdf]

Corresponding author(s): Dr. Nathalie E. Holz
Prof. Dr. Andre F. Marquand

Last updated by author(s): Jul 17, 2023

Reporting Summary

Nature Portfolio wishes to improve the reproducibility of the work that we publish. This form provides structure for consistency and transparency in reporting. For further information on Nature Portfolio policies, see our Editorial Policies and the Editorial Policy Checklist.

Statistics

For all statistical analyses, confirm that the following items are present in the figure legend, table legend, main text, or Methods section.

n/a	Confirmed	
☐	☒	The exact sample size (*n*) for each experimental group/condition, given as a discrete number and unit of measurement
☐	☒	A statement on whether measurements were taken from distinct samples or whether the same sample was measured repeatedly
☐	☒	The statistical test(s) used AND whether they are one- or two-sided<br>*Only common tests should be described solely by name; describe more complex techniques in the Methods section.*
☐	☒	A description of all covariates tested
☐	☒	A description of any assumptions or corrections, such as tests of normality and adjustment for multiple comparisons
☐	☒	A full description of the statistical parameters including central tendency (e.g. means) or other basic estimates (e.g. regression coefficient) AND variation (e.g. standard deviation) or associated estimates of uncertainty (e.g. confidence intervals)
☐	☒	For null hypothesis testing, the test statistic (e.g. *F*, *t*, *r*) with confidence intervals, effect sizes, degrees of freedom and *P* value noted<br>*Give P values as exact values whenever suitable.*
☐	☒	For Bayesian analysis, information on the choice of priors and Markov chain Monte Carlo settings
☒	☐	For hierarchical and complex designs, identification of the appropriate level for tests and full reporting of outcomes
☐	☒	Estimates of effect sizes (e.g. Cohen's *d*, Pearson's *r*), indicating how they were calculated

Our web collection on statistics for biologists contains articles on many of the points above.

Software and code

Policy information about availability of computer code

Data collection	MRI data acquired with Siemens Trio, Siemens Prisma Fit
Data analysis	Normative Modelling of adversity with Predictive Clinical Neuroscience toolkit (PCNtoolkit) software v 0.19 (https://pcntoolkit.readthedocs.io/en/ latest) implemented in python 3.6<br>Normative Modelling of age trajectories with Predictive Clinical Neuroscience toolkit (PCNtoolkit) software v 0.26 (https://pcntoolkit.readthedocs.io/en/ latest) implemented in python 3.8.0<br>PCA: sklearn package (0.24.2) implemented in python 3.6<br>preprocessing of imaging data fsl/6.0.5<br>linear mixed models lme4 (v1.1.27.1), lmerTest (v3.1.3), sjPlot (v2.8.12) packages implemented in R-4.1.0

For manuscripts utilizing custom algorithms or software that are central to the research but not yet described in published literature, software must be made available to editors and reviewers. We strongly encourage code deposition in a community repository (e.g. GitHub). See the Nature Portfolio guidelines for submitting code & software for further information.

Data

Policy information about availability of data

All manuscripts must include a data availability statement. This statement should provide the following information, where applicable:

- Accession codes, unique identifiers, or web links for publicly available datasets
- A description of any restrictions on data availability
- For clinical datasets or third party data, please ensure that the statement adheres to our policy

Data supporting the findings of this study (i.e the MARS and IMAGEN samples) are available upon reasonable request from the corresponding authors. Publicly available data derived from the lifespan normative models are available via the repositories contributing the data (Cam-CAN https://www.cam-can.org/index.php?content=dataset; PNC https://www.nitrc.org/projects/pnc; UKB https://www.ukbiobank.ac.uk, application id 23668; OASIS https://www.oasis-brains.org; HCP https://www.humanconnectome.org/study/hcp-young-adult).

Human research participants

Policy information about studies involving human research participants and Sex and Gender in Research.

Reporting on sex and gender	sex was considered in all analyses as covariate<br>information on sex was assessed at the start of the longitudinal studies (approx. 1986 & 2000) via self-report.
Population characteristics	MARS T1: 25 years (59% females), T2: 33-34 years (61 % females), IMAGEN: 22 years (57 % females)
Recruitment	Depending on pregnancy and birth history and on family background, infants were assigned to 1 of 9 groups of a 2-factorial design with factor I representing the degree of biological risk (obstetric complications) and factor II the degree of psychosocial risk (no, moderate, or high risk) (more information in the supplement). All groups had about equal size, with a slight oversampling in the high-risk combinations and with sex evenly distributed in all subgroups. A total of 384 infants born between February 1, 1986, and February 28, 1988, were recruited from 2 obstetric and 6 children's hospitals of the Rhine-Neckar region of Germany. To control confounding effects of family environment and infant medical status, only firstborn singletons of German speaking parents with no severe physical handicaps, obvious genetic defects, or metabolic diseases were selected. Participation rate at the time of recruitment was 64.5%, with a slightly lower rate in parents from psychosocially disadvantaged backgrounds. All families were Caucasians.
Ethics oversight	The study was approved by the ethics committee of the University of Heidelberg (both for MARS and IMAGEN). Written informed consent was obtained from all participants and they received monetary compensation for their involvement. Ethical approval for the public data were provided by the relevant local research authorities for the studies contributing data. For full details see the main study publications given in the supplement.

Note that full information on the approval of the study protocol must also be provided in the manuscript.

Field-specific reporting

Please select the one below that is the best fit for your research. If you are not sure, read the appropriate sections before making your selection.

☐ Life sciences ☒ Behavioural & social sciences ☐ Ecological, evolutionary & environmental sciences

For a reference copy of the document with all sections, see nature.com/documents/nr-reporting-summary-flat.pdf

Behavioural & social sciences study design

All studies must disclose on these points even when the disclosure is negative.

Study description	Mannheim Study of Children at Risk, an ongoing epidemiological at-risk cohort, following its participants since birth (1986)<br>IMAGEN, population-based study, subsample with similar sociodemographic information as in the Mannheim Study of Children at Risk
Research sample	MARS T1: n=169, 25 years (59% females), T2: n=114, 33-34 years (61 % females); sample chosen because of prospective assessments of adversity<br>IMAGEN: n=115, 22 years (57 % females); sample chosen as replication because of similar demographics and adversity assessments<br><br>publicly available data sets:<br>Philadelphia Neurodevelopmental Cohort (PNC) (Satterthwaite et al., 2014), n=1296, 8-21 y., 52% females<br>Cam-CAN (Shafto et al., 2014), n=656, 18-89 years, 51% females<br>Human Connectome Project (Van Essen et al., 2013), n=1112, 22-37 years, 55% females<br>UK Biobank (Miller et al., 2016; Sudlow et al., 2015), application id 23668, 11025.0: n=12008, 44-88 years, 52% females, 11027.0:

n=2145, 47-88 years, 55% females
OASIS3(Marcus et al., 2010), n=2144, 43-97 years, 57% females

Sampling strategy

MARS: All groups had about equal size, with a slight oversampling in the high-risk combinations and with sex evenly distributed in all subgroups (purposive sampling)
given longitudinal data, no case control design, and the Bayesian approach used that renders deviations harder to detect in smaller samples and the focus on variability at the individual level, no power calculation was performed/neccessary. The sample size of the adversity model is in line with previous studies using Gaussian models (Holz et al. 2022)

Data collection

MARS: At the 25-year-assessment, we acquired 1x1x1 mm T1-weighted anatomical images with 192 slices covering the whole brain (matrix 256x256, repetition time=2300ms, echo time=3.03ms, 50% distance factor, field of view 256x256x192mm, flip angle 9°) using a 3T scanner (Magnetom TRIO, Siemens, Erlangen, Germany) with a standard 12-channel head coil. At the 33-year-assessment high-resolution anatomical images with 208 slices covering the whole brain were acquired using a 3T-scanner (PrismaFit, Siemens) with a 32-channel head coil.
Researchers were not blinded in terms of the participants risk group during data collection. In the majority of interviews conducted during the survey when the infants were 3 months old, the interviewer and the respondent (60%), particularly the mother (95%), were present. Similarly, the subsequent interviews primarily took place between the interviewer and the parent being interviewed (70-82%).
Maternal smoking during pregnancy. Was determined by a standardized interview with the mother conducted at the 3-month assessment and classified as nonsmokers, smoking 1-5 cigarettes per day (cig./d) and more than 5 cigarettes per day for further details see 1.
Prenatal maternal stress. A standardized parent interview was conducted at the 3-month assessment. 11 questions were asked concerning worries, mood problems, as well as positive experiences during pregnancy. Mothers were requested to judge separately for the first and the second/third trimesters. As associations of prenatal stress in mid- and late pregnancy with behavioral outcome in the offspring have been reported to be largest 2, only prenatal stress during the second and third trimester was included.
Early mother-child interaction. As described in Holz et al. 3, videotapes of a 10-min standardized nursing and play situation between mothers and their three-month-olds at our lab were recorded and evaluated by trained raters (κ>0.83) using a modified version of the category system for micro-analysis of the early mother–child interaction 4,5. Raters were blind to parental and child risk status. Nine measures of mother–infant interaction behavior were formed by coding a behavior as present or absent in a total of 120 five-second intervals. Maternal stimulation included all attempts to attract the infant´s attention or to establish contact with him/her (vocal, facial or motor) and was coded when the baby was gazing at the mother or when the behaviors were clearly directed at the child. The scores were z-transformed and recoded such that higher scores represent lower stimulation.
Obstetric adversity. At the age of 3 months, an obstetric adversity score was obtained by counting the presence of 9 adverse conditions during pregnancy, delivery, and postnatal period such as preterm labor, asphyxia, or seizures. See supplemental Figure 9 for its composition.
Psychosocial adversity. Information on adverse characteristics of the parents (low educational level, broken home history or delinquency, poor coping skills, psychopathology), their partnership (early parenthood, one-parent family, unwanted pregnancy, marital discord) and the family environment (overcrowding, poor social integration and support, severe chronic life difficulties) was assessed according to an ‘enriched’ family adversity index 6 by a standardized parent interview conducted at each assessment (n=5) until the age of 11 years (range 0-9, M=2.95; SD=2.05). The score is created such that events that reflect only one possible exposure during lifetime (e.g. unwanted pregnancy) are also only counted once.
Childhood trauma. At the age of 23, participants completed the brief screening version of the Childhood Trauma Questionnaire (CTQ, Bernstein et al. 2003). The CTQ entails a retrospective assessment of five types of self-reported childhood maltreatment, i.e. sexual, physical, and emotional abuse, and emotional and physical neglect. The scores of all subscales were summed up.
Life events. To assess exposure to life stress (LS) across the life span, a semi-structured parent interview was conducted until the age of 15 years. The young adults were interviewed from the age of 19 years onwards. The interview, which was a modified and shortened version of the Munich Events List 7, evaluated the occurrence of adverse life events during a period of one year prior to the assessment. The items covered all relevant areas of children's and young adults' LS, including family, school, parents, health, legal troubles, and living conditions, such as birth of a sibling, death of a close relative or parents` separation for which the participant indicated a subjective burden. A composite score was computed by summing up the z-standardized scores from the ten assessments between the age of 3 months and 25 years.
Adult psychopathology. The Young Adult Self-Report (YASR)44 and the Adult Self-Report (ASR)45 were used to measure clinical symptoms on the basis of DSM-IV criteria at the ages of 25 and 33/34 years, respectively.

IMAGEN
Anatomical images. MRI was performed on a 3T scanner (Siemens Trio). High-resolution anatomical MR images were obtained using a standardized 3D T1-weighted magnetisation prepared rapid acquisition gradient echo (MPRAGE) sequence based on the ADNI protocol (http://adni.loni.usc.edu/ methods/mri-analysis/mri-acquisition/). The parameters were as follows: repetition time = 2300 ms, echo time = 2.93 ms, flip angle = 9̊, 1.1x1.1x1.1 mm voxel size.
Assessments. During all 4 assessment waves (14, 16, 19, 22 years), the participants completed the Life Events Questionnaire (LEQ) 10 that was adapted for their age.
The CTQ was assessed at the age of 19 years. The total score across all scales was used (mean=31.68., SD=7.64, range=25-73).

Timing

MARS: 1986-2020
IMAGEN: 2014-2020

Data exclusions

MARS: Out of 309 participants (80% of the original sample) participating in the 25-year assessment, a subsample took part in the neuroimaging session (N=200, T1). After exclusion due to left-handedness or somatic diseases (n=19), technical artefacts in the scans (n=2) and missing data (n=10), 169 healthy participants were included (58% females). Of those, 118 were scanned again at the age of 33/34 years (T2), of which 4 had to be excluded due to technical artifacts.
IMAGEN: 122 participants took part in the 22y assessment, 7 had to excluded due to missing data in adversities (n=6) and for missing brain data due to technical problems (n=1)

Philadelphia Neurodevelopmental Cohort (PNC), Cam-CAN, Human Connectome Project, UK Biobank and OASIS3 scans excluded in case of obvious image artefacts and using the Euler Characteristic criteria such as previoulsy done (Rutherford et al., 2022)

Non-participation	MARS: participation rate 80% from the original recruited sample in 1986<br>IMAGEN: participation rate 60% rom the original recruited sample in 2014
Randomization	not allocated to experimental groups.

Reporting for specific materials, systems and methods

We require information from authors about some types of materials, experimental systems and methods used in many studies. Here, indicate whether each material, system or method listed is relevant to your study. If you are not sure if a list item applies to your research, read the appropriate section before selecting a response.

Materials & experimental systems

n/a	Involved in the study
☒	☐ Antibodies
☒	☐ Eukaryotic cell lines
☒	☐ Palaeontology and archaeology
☒	☐ Animals and other organisms
☒	☐ Clinical data
☒	☐ Dual use research of concern

Methods

n/a	Involved in the study
☒	☐ ChIP-seq
☒	☐ Flow cytometry
☐	☒ MRI-based neuroimaging

Magnetic resonance imaging

Experimental design

Design type	MPRAGE
Design specifications	n.a.
Behavioral performance measures	n.a.

Acquisition

Imaging type(s)	structural
Field strength	3T
Sequence & imaging parameters	1x1x1 mm T1-weighted anatomical images with 192 slices covering the whole brain (matrix 256x256, repetition time=2300ms, echo time=3.03ms, 50% distance factor, field of view 256x256x192mm, flip angle 9°) using a 3T scanner (Magnetom TRIO, Siemens, Erlangen, Germany) with a standard 12-channel head coil<br><br>Imaging parameters for publicly available data sets as mentioned in the main papers:<br>Philadelphia Neurodevelopmental Cohort (PNC) (Satterthwaite et al., 2014)<br>Cam-CAN (Shafto et al., 2014)<br>Human Connectome Project (Van Essen et al., 2013)<br>UK Biobank (Miller et al., 2016; Sudlow et al., 2015)<br>OASIS3(Marcus et al., 2010)
Area of acquisition	whole brain

Diffusion MRI ☐ Used ☒ Not used

Preprocessing

Preprocessing software	fsl/6.0.5
Normalization	Preprocessing of the anatomical images entailed the following steps. First, images were reoriented to the standard (MNI) orientation [fslreorient2std], automatically cropped [robustfov] and bias-field corrected (RF/B1-inhomogeneity-correction) [FAST]. Then registered to standard space (linear and non-linear) [FLIRT and FNIRT], followed by brain-extraction [FNIRT-based or BET] as well as tissue-type segmentation [FAST] and subcortical structure segmentation [FIRST].<br>The JD images were affine and log transformed and masked by a grey matter template.<br><br>In addition, we estimated the normative model on grey matter density, which we calculated by warping grey matter to MNI space and subsequent multiplication by the jacobian determinants and smoothing with a 6mm kernel.

Normalization template | MNI152_T1_1mm.nii

Noise and artifact removal | Data were visually inspected and evaluated by an experienced rater (NH).

Volume censoring | n.a.

Statistical modeling & inference

Model type and settings | Bayesian Linear Regression under 10-fold crossvalidation; Bayesian Linear Regression (BLR) with likelihood warping ('sinarcsinsh' warping function); linear mixed models

Effect(s) tested | contraction or expansion of deformation fields as a function of lifetime adversities exposure profiles, sex and total intracranial volume
contraction or expansion of JDs as a function of age, sex and site
prediction of anxiety based on individual deviations from the normative model

Specify type of analysis: ☒ Whole brain ☐ ROI-based ☐ Both

Statistic type for inference (See Eklund et al. 2016) | voxel-wise

Correction | Bonferroni for association with psychopathology; 10-fold crossvalidation

Models & analysis

n/a	Involved in the study
☐	☐ Functional and/or effective connectivity
☐	☐ Graph analysis
☐	☒ Multivariate modeling or predictive analysis

Functional and/or effective connectivity | n.a.

Graph analysis | n.a.

Multivariate modeling and predictive analysis | A model of normative deformation field development as a function of adversity and sex was created by training a Bayesian Linear Regression (BLR) model using the Predictive Clinical Neuroscience toolkit (PCNtoolkit) software (https://pcntoolkit.readthedocs.io/en/ latest) implemented in python 3.6. In our BLR analysis predictions are derived in an unbiased manner under 10-fold cross-validation. Briefly, this Bayesian approach calculates the probability distribution over all functions that fit the data while specifying a prior over all possible values and relocating probabilities based on evidence (i.e. observed data). As such, it yields unbiased estimates of generalizability and inferences with increasing uncertainty with fewer data. This, in turn, increases the conservativeness of this approach and renders deviations harder to detect.
The accuracy of the reference model showing the long-term adversity signature was evaluated using the correlation between the true and the predicted voxel values (Rho). Structure coefficients, that reflect the correlation between a predictor and the expected outcome, were calculated to assess the contribution of each single adversity to the predicted model.
To estimate a pattern of regional deviations from typical brain structure for each participant, we derived normative probability maps (NPM) that quantify the voxel-wise deviation from the normative model. This was done by calculating an individual-specific Z score21 indicating the difference between the prediction (mean, ŷij) at each brain location (j) and true brain structure (yij) scaled by the prediction variance [expected level of variation σ2ij and variance learned from the normative distribution (σ2nj)

Age model
Normative modeling was run using python 3.8 and the PCNtoolkit package (version 0.26). Bayesian Linear Regression (BLR) with likelihood warping ('sinarcsinsh' warping function)20,21 was used to model voxel-wise JD development from a vector of covariates (age, sex, and site) . For each voxel y is predicted as:
y=wTϕ(x)+ϵ
where wT is the estimated weight vector, ϕ(x) is a basis expansion of the of covariate vector x, consisting of a B-spline basis expansion (cubic spline with five evenly spaced knots) to model non-linear effects of age, and ϵ=N(0,β-1) a Gaussian noise distribution with mean zero and noise precision term β (the inverse variance).
